# Prevalence and outcomes of co-infection and superinfection with SARS-CoV-2 and other pathogens: A systematic review and meta-analysis

**DOI:** 10.1371/journal.pone.0251170

**Published:** 2021-05-06

**Authors:** Jackson S. Musuuza, Lauren Watson, Vishala Parmasad, Nathan Putman-Buehler, Leslie Christensen, Nasia Safdar

**Affiliations:** 1 Division of Infectious Disease, Department of Medicine, University of Wisconsin School of Medicine and Public Health, Madison, WI, United States of America; 2 William S. Middleton Memorial Veterans Hospital, Madison, WI, United States of America; 3 Ebling Library for the Health Sciences, University of Wisconsin School of Medicine and Public Health, Madison, WI, United States of America; University of South Dakota, UNITED STATES

## Abstract

**Introduction:**

The recovery of other pathogens in patients with SARS-CoV-2 infection has been reported, either at the time of a SARS-CoV-2 infection diagnosis (co-infection) or subsequently (superinfection). However, data on the prevalence, microbiology, and outcomes of co-infection and superinfection are limited. The purpose of this study was to examine the occurrence of co-infections and superinfections and their outcomes among patients with SARS-CoV-2 infection.

**Patients and methods:**

We searched literature databases for studies published from October 1, 2019, through February 8, 2021. We included studies that reported clinical features and outcomes of co-infection or superinfection of SARS-CoV-2 and other pathogens in hospitalized and non-hospitalized patients. We followed PRISMA guidelines, and we registered the protocol with PROSPERO as: CRD42020189763.

**Results:**

Of 6639 articles screened, 118 were included in the random effects meta-analysis. The pooled prevalence of co-infection was 19% (95% confidence interval [CI]: 14%-25%, I^2^ = 98%) and that of superinfection was 24% (95% CI: 19%-30%). Pooled prevalence of pathogen type stratified by co- or superinfection were: viral co-infections, 10% (95% CI: 6%-14%); viral superinfections, 4% (95% CI: 0%-10%); bacterial co-infections, 8% (95% CI: 5%-11%); bacterial superinfections, 20% (95% CI: 13%-28%); fungal co-infections, 4% (95% CI: 2%-7%); and fungal superinfections, 8% (95% CI: 4%-13%). Patients with a co-infection or superinfection had higher odds of dying than those who only had SARS-CoV-2 infection (odds ratio = 3.31, 95% CI: 1.82–5.99). Compared to those with co-infections, patients with superinfections had a higher prevalence of mechanical ventilation (45% [95% CI: 33%-58%] vs. 10% [95% CI: 5%-16%]), but patients with co-infections had a greater average length of hospital stay than those with superinfections (mean = 29.0 days, standard deviation [SD] = 6.7 vs. mean = 16 days, SD = 6.2, respectively).

**Conclusions:**

Our study showed that as many as 19% of patients with COVID-19 have co-infections and 24% have superinfections. The presence of either co-infection or superinfection was associated with poor outcomes, including increased mortality. Our findings support the need for diagnostic testing to identify and treat co-occurring respiratory infections among patients with SARS-CoV-2 infection.

## Introduction

The coronavirus disease 2019 (COVID-19) pandemic is associated with high morbidity and mortality [1, 2]. Current evidence shows that severe acute respiratory syndrome coronavirus 2 (SARS-CoV-2), the causative agent of COVID-19, is primarily transmitted through respiratory droplets [3, 4] from symptomatic, asymptomatic, or pre-symptomatic individuals [4, 5]. Similar to other respiratory pathogens, such as influenza, where approximately 25% of older patients get secondary bacterial infections [6, 7], both superinfections and co-infections with SARS-CoV-2 have been reported [8–10]. However, there is scarce data on the frequency of co-infection and superinfections by viral, bacterial, or fungal infections and associated clinical outcomes among patients infected with SARS-CoV-2 [8–10].

We define co-infection as the recovery of other respiratory pathogens in patients with SARS-CoV-2 infection at the time of a SARS-CoV-2 infection diagnosis and superinfection as the subsequent recovery of other respiratory pathogens during care for SARS-CoV-2 infection. Two previous reviews have examined the prevalence of bacterial and fungal co-infection or superinfection in SARS-CoV-2 infected patients [11, 12]. In addition, prior work suggests outcome differences in patients with co-infections vs. superinfections. For example, Garcia-Vidal et al., showed that SARS-CoV-2 infected patients with superinfection s had a longer length of hospital stay (LOS) and higher mortality, while those with co-infections had a higher frequency of admission to the ICU [13].

Diagnostic testing and therapeutic decision-making may be affected by the presence of co-infection or superinfection with SARS-CoV-2 and other respiratory pathogens.

Therefore, we conducted a systematic review and meta-analysis to examine the occurrence and outcomes (e.g., LOS) of respiratory co-infections and superinfections among patients infected with SARS-CoV-2.

## Materials and methods

We conducted this systematic review in accordance with the Preferred Reporting in Systematic Reviews and Meta-Analyses (PRISMA) guidelines [14]. We registered this review with PROSPERO: CRD42020189763 [15]. The protocol is available as a S1 File.

### Data sources and searches

With the help of a health sciences librarian (LC), we searched PubMed, Scopus, Wiley, Cochrane Central Register of Controlled Trials, Web of Science Core Collection, and CINAHL Plus databases to identify English-language studies published from October 1, 2019, through February 8, 2021. We executed the search in PubMed and translated the keywords and controlled vocabulary for the other databases, and additional articles were added from reference lists of pertinent articles. The following keywords were used for the search: “coronavirus”,”coronavirus infections”, “HCoV”, “nCoV”, “Covid”, “SARS”, "COVID-19", “2019 nCoV”, “nCoV 19”, “SARS-CoV-2”, “SARS coronavirus2”, “2019 novel corona virus”, “Human”, “pneumonia”, “influenza”, “severe acute respiratory syndrome”, “co-infection”, “Superinfection”, “bacteria”, “fungus”, “concomitant”, “pneumovirinae”, “pneumovirus infections”, "respiratory syncytial viruses", “metapneumovirus”, “influenza”, “human”, “respiratory virus”, “bacterial Infections”, “viral infection”, “fungal infection”, “upper respiratory”, “oxygen inhalation therapy”, “intensive care units”, “nursing homes”, “subacute care”, “skilled nursing”, “intermediate care”, “patient discharge”, “mortality”, “morbidity” and English filter. A complete description of our search strategy is available as a S2 File.

### Study selection

Citations were uploaded into Covidence®, an online systematic review software for the study selection process. Two authors (JSM and LW) independently screened titles and abstracts and read the full texts to assess if they met the inclusion criteria. The authors met and discussed any articles where there was conflict and decided to either include or exclude such articles. Inclusion criteria were randomized clinical trials (RCTs), quasi-experimental and observational human studies that reported clinical features and outcomes of co-infection or superinfection of SARS-CoV-2 (laboratory-confirmed) and other pathogens–fungal, bacterial, or other viruses–in hospitalized and non-hospitalized patients. We excluded studies that did not report co-infection or superinfection, editorials, reviews, qualitative studies, those published in a non-English language, articles where full texts were not available, and non-peer-reviewed preprints.

### Data extraction

Three reviewers (JSM, LW, and VP) independently abstracted data from individual studies using a standardized template. We abstracted data on study design/methodology, location and setting (intensive care unit [ICU], inpatient non-ICU, or outpatient, where applicable), study population, use of antibiotics, proportion of patients with co-infections, implicated pathogens, method of detection of co-infections and superinfections (laboratory-verified or clinical features only), type of infection (bacterial, viral, or fungal), and outcomes of co-infected patients (death, mechanical ventilation, discharge disposition, length of hospital stay, or mild illness). Discrepancies were resolved by discussion between the three abstractors.

### Risk of bias assessment

Risk of bias assessment was conducted by three authors (JSM, LW, and VP) independently. We used two study quality assessment tools, one specific to case series [16], and one for non-case series study designs [17].

The tool for case series examines four domains: selection, ascertainment, causality, and reporting [16]. The selection domain helps to assess whether participants included in a study are representative of the entire population from which they arise. Ascertainment assesses whether the exposure and outcome were adequately ascertained. Causality assesses the potential for alternative explanations and specifically for our study whether the follow-up was long enough for outcomes to occur. Reporting evaluates if a study described participants in sufficient detail to allow for replication of the findings. This tool consists of eight items, but only five were applicable to our study [16]. When an item was present in a study, a score of 1 was assigned and 0 if the item was missing. We added the scores (minimum of 0 and a maximum of 5) and assigned the risk of bias as follows: low risk (5), medium risk (3–4), high risk (0–2).

For non-case series studies, we used the Modified Downs and Black risk assessment scale to assess the quality of cohort studies and RCTs [17]. This scale consists of 27 items that assess study characteristics, such as internal validity (bias and confounding), statistical power, and external validity. We scored studies as low risk (score 20–27, medium risk (score 15–19), or high risk (score ≤14).

### Data synthesis and analysis

The primary outcome was the prevalence of co-infections or superinfections by viral, bacterial, or fungal respiratory infections and SARS-CoV-2. We examined whether co-infection or superinfection was associated with an increased risk for the following patient outcomes: 1) mechanical ventilation, 2) admission to the ICU, 3) mortality and LOS.

We estimated the proportion of patients with co-infection or superinfection of viral, bacterial, and fungal respiratory infections and SARS-CoV-2. We anticipated a high level of heterogeneity given the novelty of COVID-19 and potential differences in testing and management of COVID-19 in the healthcare systems of the countries where the studies were conducted. We conducted all statistical analyses using Stata software, version 16.0 (Stata Corp. College Station, Texas). We used the “metan” and “metaprop” commands in Stata to estimate the pooled proportion of co-infection and superinfection and COVID-19 using a random effects model (DerSimonian Laird) [18, 19]. We stabilized the variance using the Freeman-Tukey arcsine transformation methodology in order to correctly estimate extreme proportions (i.e., those close to 0% or 100%) [18]. We assessed heterogeneity using the I^2^ statistic. Frequencies of outcome variables and study characteristics were estimated using descriptive statistics. For example, in studies where data on co-infecting or super-infecting pathogens were reported, we extracted and tallied the number of different pathogens reported. We calculated the proportion of pathogens using the number of pathogens as the numerator and the total number of pathogens of each type (bacteria, viruses, and fungi) from all the studies as the denominator.

We did not assess for publication bias because standard methods, such as funnel plots and associated tests, were developed for comparative studies and therefore do not produce reliable results for meta-analysis of proportions [20, 21].

## Results

**Fig 1:**
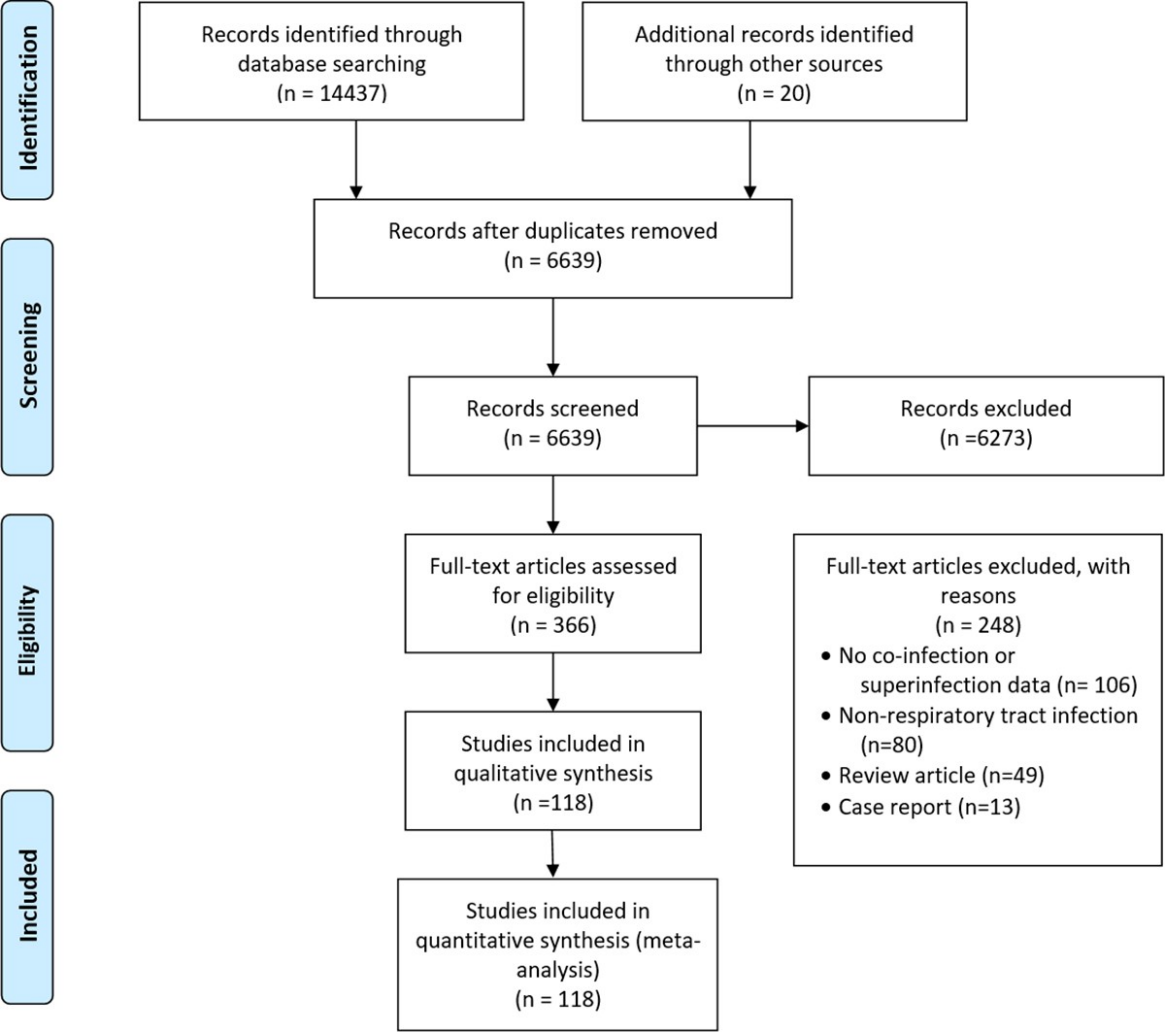
Study selection flow diagram: Adapted from the PRISMA guideline [11].

Our search yielded 14457 records; we excluded 7818 duplicates and screened 6639 articles. At the abstract and title review stage, we excluded 6273 articles, leaving 366 articles for full-text review. Of these, 118 articles met the inclusion criteria and were included in this meta-analysis. The most frequent reason for exclusion of studies at the full-text review stage was the absence of superinfection or co-infection data (Fig 1).

Approximately half of the studies (60/118) were retrospective cohort studies, 35% (42/118) were cases series, and 9% (11/118) were prospective cohort studies. There were two case-control studies, two cross-sectional studies, and one clinical trial. The majority of the studies were conducted in China (42% [49/118)]) and the US (15% [18/118]). Most of the studies were conducted in a mixed setting (i.e., ICU and non-ICU setting; 72% [85/118]) and 92% (108/118) were conducted exclusively in hospitalized patients. The majority of studies were conducted among adults (73% [86/118]). Sixty-seven (57%) of the included studies reported that patients included had co-infections, 37% (44/118) reported superinfections, and 6% (7/118) reported both co-infections and superinfections among patients. Viral co-infections in patients were reported in 67% (55/81) of the studies, bacterial infections in 74% (78/105), fungal in 48% (35/73) of studies. Not all of the 118 studies reported data on viral, bacterial or fungal infections (Table 1). Seventy percent (83/118) of the studies reported data on antibiotic use. Of these, antibiotics were administered in 98% (81/83) of the studies.

**Table 1:** Main characteristics of included studies.

Study	Study design	Country	Setting	Number of patients	Age group of patients	Gender (% male)	ICU (%)	Patients who were ventilated n (%)	Patients who died n (%)	Viral co-infections n (%)	Bacterial co-infection n (%)	Fungal co-infections n (%)	Risk of bias
Arentz, 2020 [22]	Case series	USA	ICU^a^	21	Adults	52	100	15 (71)	11 (52)	3 (14)	1 (50)	0 (0)	Medium
Barrasa, 2020 [23]	Case series	Spain	ICU	48	Adults	56	100	45 (94)	16 (33)	0 (0)	6 (13)	0 (0)	Low
Campochiaro, 2020 [24]	Prospective cohort	Italy	ICU and non-ICU	65	Adults	29	6	25 (38)	16 (25)	0 (0)	1 (2)	0 (0)	Low
Chen, 2020 [25]	Case series	China	ICU	99	Adults	68	100	17 (17)	11 (11)	0 (0)	1 (1)	4 (4)	Medium
Cuadrado-Payán, 2020 [26]	Case series	Spain	ICU	4	Adults	75	75	3 (75)	0 (0)	4 (100)	0 (0)	0 (0)	High
Ding, 2020 [27]	Case series	China	Non-ICU	115	Adults	NR^b^	0	0 (0)	0 (0)	5 (4)	0 (0)	0 (0)	Medium
Dong, 2020 [28]	Case series	China	Non-ICU	11	Adults/children	54	0	1 (9)	0 (0)	1 (9)	0 (0)	0 (0)	Medium
Du, 2020 [29]	Case series	China	ICU	109	Adults	67.9	48.6	33 (30)	109 (100)	0 (0)	NR	NR	Low
Fan, 2020 [30]	Retrospective cohort	China	ICU and non-ICU	50	Adults	83	54	23 (46)	12 (24)	0 (0)	5 (10)	5 (10)	Low
Feng, 2020 [31]	Case series	China	ICU and non-ICU	476	Adults	56.9	26	70 (15)	38 (8)	0 (0)	35 (7)	0 (0)	Medium
Garazzino, 2020 [32]	Retrospective cohort	Italy	ICU and non-ICU	168	Children	55.9	1.1	2 (1)	0 (0)	10 (6)	1 (0.5)	0 (0)	Low
Gayam, 2020 [33]	Case series	USA	ICU and non-ICU	350	Adults	33	NR	NR	NR	0 (0)	1 (0.3)	0 (0)	Medium
Huang, 2020 [34]	Case series	China	ICU and non-ICU	41	Adults	73	32	4 (10)	6 (15)	0 (0)	1 (2)	0 (0)	Medium
Kakuya, 2020 [35]	Case series	Japan	Non-ICU	3	Children	100	0 (0)	0 (0)	0 (0)	1 (33)	0 (0)	0 (0)	Low
Khodamoradi, 2020 [36]	Case series	Iran	Non-ICU	4	Adults	75	0	0 (0)	0 (0)	4 (100)	0 (0)	0 (0)	Medium
Kim, 2020 [37]	Retrospective cohort	USA	Non-ICU	115	Adults/children	45	0	0 (0)	0 (0)	25 (22)	0 (0)	0 (0)	Low
Koehler, 2020 [38]	Case series	Germany	ICU	19	Adults	NR	100	NR	3 (16)	2 (11)	0 (0)	5 (26)	Medium
Lian, 2020 [39]	Retrospective cohort	China	ICU and non-ICU	788	Children/Adults	52	3	18 (2)	0 (0)	NR	0 (0)	0 (0)	Low
Lin, 2020 [8]	Case series	China	ICU and non-ICU	92	Adults	NR	NR	NR	NR	6 (7)	NR	NR	Medium
Liu, 2020 [40]	Retrospective cohort	China	ICU and non-ICU	12	Children/Adults	66	NR	6 (50)	NR	0 (0)	2 (17)	0 (0)	Low
Lv, 2020 [41]	Retrospective cohort	China	ICU and non-ICU	354	Adults	49	NR	NR	11 (3)	1 (0.3)	32 (9)	6 (2)	Low
Ma, 2020 [42]	Retrospective cohort	China	NR	93	Adults	55	NR	NR	44 (47)	46 (49)	0 (0)	0 (0)	Low
Mannheim, 2020 [43]	Case series	USA	ICU and non-ICU	64	Children	56	11	NR	0 (0)	3 (5)	1 (2)	0 (0)	Medium
Mo, 2020 [44]	Case series	China	ICU and non-ICU	155	Adults	55	NR	36 (23)	22 (14)	13 (8)	2 (1)	0 (0)	Medium
Nowak, 2020 [9]	Case series	USA	ICU and non-ICU	1204	Adults	56	NR	NR	NR	36 (3)	0 (0)	0 (0)	Medium
Ozaras, 2020 [45]	Case series	Turkey	ICU and non-ICU	1103	Adults	50	NR	NR	NR	6 (0.5)	0 (0)	0 (0)	Medium
Palmieri, 2020 [46]	Retrospective cohort	Italy	ICU and non-ICU	3032	Children/Adults	67	NR	NR	3032 (100)	NR	NR	NR	Low
Peng, 2020 [47]	Retrospective cohort	China	ICU and non-ICU	75	Children	58	NR	NR	0 (0)	8 (11)	31 (41)	0 (0)	Low
Pongpirul, 2020 [48]	Case series	Thailand	ICU and non-ICU	11	Adults	54	NR	0 (0)	0 (0)	2 (18)	5 (45)	0 (0)	Low
Richardson, 2020 [49]	Case series	USA	ICU and non-ICU	5700	Adults	60	14.2	1151 (20)	553 (10)	39 (0.7)	3 (0.1)	0 (0)	Low
Sun, 2020 [50]	Retrospective cohort	China	ICU and non-ICU	36	Children	61	NR	NR	1 (3)	1 (3)	1 (3)	0 (0)	Medium
Tagarro, 2020 [51]	Retrospective cohort	Spain	ICU and non-ICU	41	Children	44	9.7	4 (10)	0 (0)	2 (5)	0 (0)	0 (0)	Low
Wan, 2020 [52]	Case series	China	ICU and non-ICU	135	Adults	53	NR	28 (21)	1 (0.7)	NR	NR	NR	Medium
Wang Y, 2020 [53]	Case series	China	ICU and non-ICU	55	Adults	40	0	0 (0)	0 (0)	1 (2)	1 (2)	1 (3)	Low
Wang L, 2020 [54]	Case series	China	ICU and non-ICU	339	Adults	49	NR	NR	65 (19)	0 (0)	1 (0.3)	1 (0.3)	Low
Wang R, 2020 [55]	Case series	China	ICU and non-ICU	125	Adults	56.8	15.2	4	0 (0)	1 (0.8)	9 (7)	9 (7)	Medium
Wang Y, 2020 [56]	Clinical trial	China	ICU and non-ICU	237	Adults	56	NR	21 (9)	14 (6)	NR	NR	NR	Medium
Wee, 2020 [57]	Prospective cohort	Singapore	ICU and non-ICU	3807	Adults	NR	NR	NR	1 (0.02)	3 (0.08)	NR	NR	Medium
Wu C, 2020 [58]	Retrospective cohort	China	ICU and non-ICU	201	Adults	63.7	26.4	67 (33)	44 (22)	1 (0.5)	0 (0)	0 (0)	Low
Xia, 2020 [59]	Case series	China	ICU and non-ICU	20	pediatric	65	NR	0 (0)	0 (0)	4 (0.2)	1 (5)	1 (5)	Medium
Yang X, 2020 [60]	Case series	China	ICU	710	Adults	67	100	37 (5)	32 (4)	0 (0)	4 (0.6)	4 (0.6)	Low
Yi, 2020 [61]	Case series	USA	ICU and non-ICU	132	Adult	62	50	5 (4)	1 (0.8)	NR	NR	NR	Medium
Zhang J, 2020 [62]	Case series	China	ICU and non-ICU	140	Adults	50.7	NR	NR	NR	2 (1)	1 (0.7)	1 (0.7)	Medium
Zhang G, 2020 [63]	Case series	China	ICU and non-ICU	221	Adults	48.9	80	26 (12)	5 (2)	2 (0.9)	6 (3)	6 (3)	Medium
Zhao, 2020 [64]	Case series	China	ICU and non-ICU	34	Adults	57.9	0	0 (0)	0 (0)	1 (3)	1 (3)	0 (0)	Medium
Zheng, 2020 [65]	Case series	China	ICU and non-ICU	1001	Adult and pediatric	NR	NR	NR	NR	2 (0.2)	NR	NR	Low
Zhou, 2020 [66]	Retrospective cohort	China	ICU and non-ICU	191	Adult	62	26	32 (17)	54 (28)	NR	NR	NR	Low
Zhu, 2020 [67]	Retrospective cohort	China	ICU and non-ICU	257	Adult and pediatric	53.7	1.16	0 (0)	0 (0)	9 (3)	11 (4)	11 (4)	Low
Alvares P, 2020 [68]	Retrospective cohort	Brazil	ICU and non-ICU	32	Pediatric	59.3	9.3	2 (6)	1 (3)	1 (3)	NR	NR	Medium
Borman, 2020 [69]	Case series	UK	ICU	719	Adults	NR	100.0	NR	NR	NR	NR	3NR	Low
Chauhdary W, 2020 [70]	Case series	Brunei Darussalam	ICU and non-ICU	141	Adults	NR		NR	NR	NR	7 (5)	NR	Low
Cheng L, 2020 [71]	Retrospective cohort	Hong Kong	ICU and non-ICU	147	Adults	85.0	3.0	NR	NR	NR	4 (3)	NR	Low
Cheng Y, 2020 [72]	Retrospective cohort	China	ICU and non-ICU	213	Adults	50.2		2 (1)	8 (4)	97 (46)	NR	NR	Low
Cheng K, 2020 [73]	Retrospective cohort	China	NR	212	Adults/Children	51.0		19 (9)	NR	NR	13 (6)	NR	Low
Contou D, 2020 [74]	Retrospective cohort	France	ICU	92	Adults	79.0	100.0	83 (90)	45 (49)	NR	32 (35)	NR	Low
Dupont D, 2020 [75]	Case series	France	ICU	19	Adults	78.0	100.0	18 (95)	NR	NR	NR	19 (100)	Low
Elabbadi A, 2020 [76]	Case series	France	ICU	101	Adults	78.2	100.0	83 (82)	21 (21)	NR	10 (10)	NR	Low
Falces-Romero, 2020 [77]	Retrospective cohort	Spain	ICU and non-ICU	10	Adults	80.0	70.0	7 (70)	7 (70)	NR	0	10 (100)	Medium
Falcone M, 2020 [78]	Prospective cohort	Italy	ICU and non-ICU	315	Adults	66.6	26.9	55 (17)	70 (22)	NR	11 (3)	2 (1)	Medium
Fu Y, 2020 [79]	Case series	China	ICU and non-ICU	5	Adults	80.0	100.0	5 (100)	NR	NR	5 (100)	2 (40)	Low
Garcia-Menino, 2021 [80]	Case series	Spain	ICU	7	Adults	86.0	100.0	NR	1 (14)	NR	7 (100)	NR	Low
Garcia-Vidal, 2021 [81]	Prospective cohort	Spain	ICU and non-ICU	989	Adults	55.8	15.0	NR	103 (10)	6 (1)	47 (5)	7 (1)	Low
Gouzien, 2020 [82]	Retrospective cohort	France	ICU	53	Adults	67.9	100.0	53 (100)	39 (74)	NR	NR	1 (2)	Medium
Hashemi S, 2020 [83]	Case series	Iran	ICU and non-ICU	105	Adults/Children	NR		NR	105 (100)	NR	NR	NR	Low
Hazra A, 2020 [84]	Retrospective cohort	USA	ICU and non-ICU	459	NR	NR		NR	NR	6 (1)	NR	NR	High
He Bing, 2020 [85]	Retrospective cohort	China	NR	21	Adults/Children	NR		NR	0	NR	2 (10)	4 (19)	Medium
Hirotsu Y, 2020 [86]	Prospective cohort	Japan	non-ICU	191	NR	NR		NR	NR	32 (17)	NR	NR	Medium
Hughes, 2020 [87]	Case series	UK	ICU	836	Adults	62.0		NR	262 (31)	NR	5 (1)	27 (3)	Low
Karaba, 2020 [88]	Retrospective cohort	USA	ICU and non-ICU	1016	Adults	54.0	12.0	NR	NR	2 NR	1NR	NR	Low
Kolenda, 2020 [89]	Prospective cohort	France	ICU	99	NR	NR	100.0	NR	NR	NR	17 (17)	NR	Low
Kumar, 2021 [90]	Retrospective cohort	USA	ICU and non-ICU	1573	Adults	57.9	31.0	247 (16)	413 (26)	NR	48 (3)	9 (1)	Low
Lardaro T, 2020 [91]	Retrospective cohort	USA	ICU and non-ICU	542	Adults	49.6	15.9	159 (29)	78 (14)	NR	8 (1)	NR	Medium
Lehmann C, 2020 [92]	Retrospective cohort	USA	ICU and non-ICU	321	Adults	48.0	5.0	NR	22 (7)	5 (2)	7 (2)	NR	Medium
Lendorf, 2020 [93]	Retrospective cohort	Denmark	ICU and non-ICU	115	Adults/Children	60.0	18.0	12 (10)	16 (14)	NR	9 (8)	1 (1)	Medium
Li J, 2020 [94]	Retrospective cohort	China	ICU and non-ICU	102	Adults/Children	66.7		NR	50 (49)	NR	159 (156)	NR	Medium
Li Z, 2020 [95]	Retrospective cohort	China	ICU and non-ICU	32	Adults	62.5	40.0	6 (19)	NR	6 (19)	10 (31)	2 (6)	High
Ma L, 2020 [96]	Retrospective cohort	China	ICU and non-ICU	250	Adults	46.0		5 (2)	4 (2)	4 (2)	2 (1)	NR	Low
Mahmoudi H, 2020 [97]	Cross-sectional study	Iran	ICU and non-ICU	342	Adults	NR		NR	NR	NR	6 (2)	NR	Medium
Mendes N, 2020 [98]	Retrospective cohort	USA	ICU and non-ICU	242	Adults	50.8		54 (22)	52 (21)	NR	6 (2)	NR	Low
Mughal, 2020 [99]	Restrospective cohort	USA	ICU and non-ICU	129	Adults	62.8	30.2	30 (23)	20 (16)	NR	NR	NR	Low
Nasir N, 2020 [100]	Retrospective cohort	Pakistan	ICU and non-ICU	30	Adults	83.0	33.0	24 (80)	7 (23)	NR	6 (20)	7 (23)	Low
Nasir N, 2020 [101]	Retrospective cohort	Pakistan	ICU and non-ICU	147	Adults	60.0			NR	NR	9 (6)	1 (1)	Medium
Ng K F, 2020 [102]	Case series	China	ICU and non-ICU	8	Pediatric	25.0	25.0	NR	NR	5 (63)	NR	NR	Low
Nori, 2021 [103]	Retrospective cohort	USA	ICU and non-ICU	152	Adults/Children	59.0	55.9	NR	86 (57)	NR	112 (74)	3 (2)	Low
Obata, 2020 [104]	Retrospective cohort	USA	ICU and non-ICU	226	Adults	55.1	24.8	NR	41 (18)	NR	8 (4)	8 (4)	Medium
Oliva, 2020 [105]	Case series	Italy	ICU and non-ICU	7	Adults	57.0	14.3	NR	NR	NR	7 (100)	NR	Low
Papamanoli, 2020 [106]	Retrospective cohort	USA	ICU and non-ICU	447	Adults	66.0	45.2	115 (26)	102 (23)	NR	NR	NR	Low
Peci A, 2021 [107]	Case-control	Canada	ICU and non-ICU	325	Adults/Children	NR		NR	NR	8 (2)	NR	NR	Low
Pereira, 2021 [108]	Case-control	New York	ICU and non-ICU	87	Adults	60.9	48.3	NR	32 (37)	10 (11)	6 (7)	1 (1)	Medium
Pettit, 2020 [109]	Retrospective cohort	USA	ICU and non-ICU	148	Adults	37.5	70.3	48 (32)	46 (31)	1 (1)	14 (9)	2 (1)	Low
Pickens, 2021 [110]	Retrospective cohort	Chicago	ICU	179	Adults	61.5	100.0	179 (100)	34 (19)	NR	28 (16)	NR	Low
Ramadan H, 2021 [111]	Prospective cohort	Egypt	ICU and non-ICU	260	Adults	55.4		8 (3)	24 (9)	NR	37 (14)	NR	Low
Reig S, 2020 [112]	Retrospective cohort	Germany	ICU and non-ICU	213	Adults	61.0	33.0	57 (27)	51 (24)	NR	26 (12)	6 (3)	Low
Ripa M, 2020 [113]	Prospective cohort	Italy	ICU and non-ICU	731	Adults	68.0	12.0	NR	194 (27)	NR	24 (3)	11 (2)	Low
Rothe K, 2020 [114]	Retrospective cohort	Germany	ICU and non-ICU	140	Adults	64.0	15.0	41 (29)	NR	NR	NR	9 (6)	Low
Segrelles-Calvo G, 2021 [115]	Case series	Spain	ICU and non-ICU	7	Adults	71.0	86.0	7 (100)	5 (71)	NR	NR	7 (100)	Low
Sharifipour E, 2020 [116]	Prospective cohort	Iran	ICU	19	Adults	58.0	100.0	19 (100)	18 (95)	NR	19 (100)	NR	Low
Sogaard, 2021 [117]	Retrospective cohort	Switzerland	ICU and non-ICU	162	Adults	61.1	25.3	NR	17 (10)	5 (3)	19 (12)	3 (2)	Low
Soriano, 2021 [118]	Retrospective cohort	Spain	ICU	83	Adults	79.0	100.0	78 (94)	20 (24)	NR	7 (8)	NR	Low
Tang, 2021 [119]	Retrospective cohort	China	NR	78	Adults/Children	53.0		NR	NR	4 (5)	5 (6)	NR	Low
Torrego, 2020 [120]	Retrospective cohort	Spain	ICU	163	NR	NR	100.0	139 (85)	23 (14)	NR	18 (11)	NR	High
Townsend, 2020 [121]	Prospective cohort	Ireland	ICU and non-ICU	117	Adults	63.0	29.1	NR	17 (15)	NR	6 (5)	1 (1)	Low
Verroken, 2020 [122]	Prospective cohort	Belgium	ICU	32	NR	NR	100.0	NR	NR	NR	13 (41)	NR	Medium
Wang L, 2020 [123]	Retrospective cohort	UK	ICU and non-ICU	1396	Adults	65.0	30.0	NR	420 (30)	NR	11 (1)	NR	Low
Wei L, 2020 [124]	Retrospective cohort	China	non-ICU	43	Adults	0.0	0.0	NR	NR	15 (35)	NR	NR	Low
White P, 2020 [125]	Retrospective cohort	UK	ICU and non-ICU	135	Adults	69.0		NR	51 (38)	NR	NR	36 (27)	Low
Wu Q, 2020 [126]	Retrospective cohort	China	NR	74	Pediatric	59.5		1 (1)	NR	10 (14)	16 (22)	NR	Low
Xia P, 2020 [127]	Retrospective cohort	China	ICU	81	Adults	66.7	100.0	66 (81)	60 (74)	NR	34 (42)	NR	Low
Xu J, 2020 [128]	Retrospective cohort	China	ICU	239	Adults	59.8	100.0	165 (69)	147 (62)	NR	25 (10)	NR	Low
Xu S, 2020 [129]	Retrospective cohort	China	ICU and non-ICU	64	Adults	0.0	1.6	NR	NR	9 (14)	10 (16)	NR	Low
Xu W, 2021 [130]	Retrospective cohort	China	ICU and non-ICU	659	Adults/Children	50.4	5.0	NR	NR	NR	48 (7)	NR	Low
Yao T, 2020 [131]	Retrospective cohort	China	NR	83	Adults	63.9		71 (86)	83 (100)	NR	36 (43)	NR	Low
Yu C, 2020 [132]	Retrospective cohort	China	NR	128	Adults	43.0		NR	14 (11)	64 (50)	5 (4)	NR	Low
Yue H, 2020 [133]	Retrospective cohort	China	NR	307	Adults	47.3		NR	NR	176 (57)	NR	NR	Medium
Yusuf E, 2021 [134]	Case-control	Netherlands	ICU	92	Adults	76.1	100.0	NR	NR	NR	NR	10 (11)	High
Zhang C, 2020 [135]	Retrospective cohort	China	NR	34	Pediatric	41.0		NR	NR	13 (38)	9 (26)	NR	Low
Zhang H, 2020 [136]	Retrospective cohort	China	NR	38	Adults	84.2		23 (61)	8 (21)	NR	37 (97)	3 (8)	Low

^a^ICU: intensive care unit.

^b^NR: Not reported.

**Fig 2:**
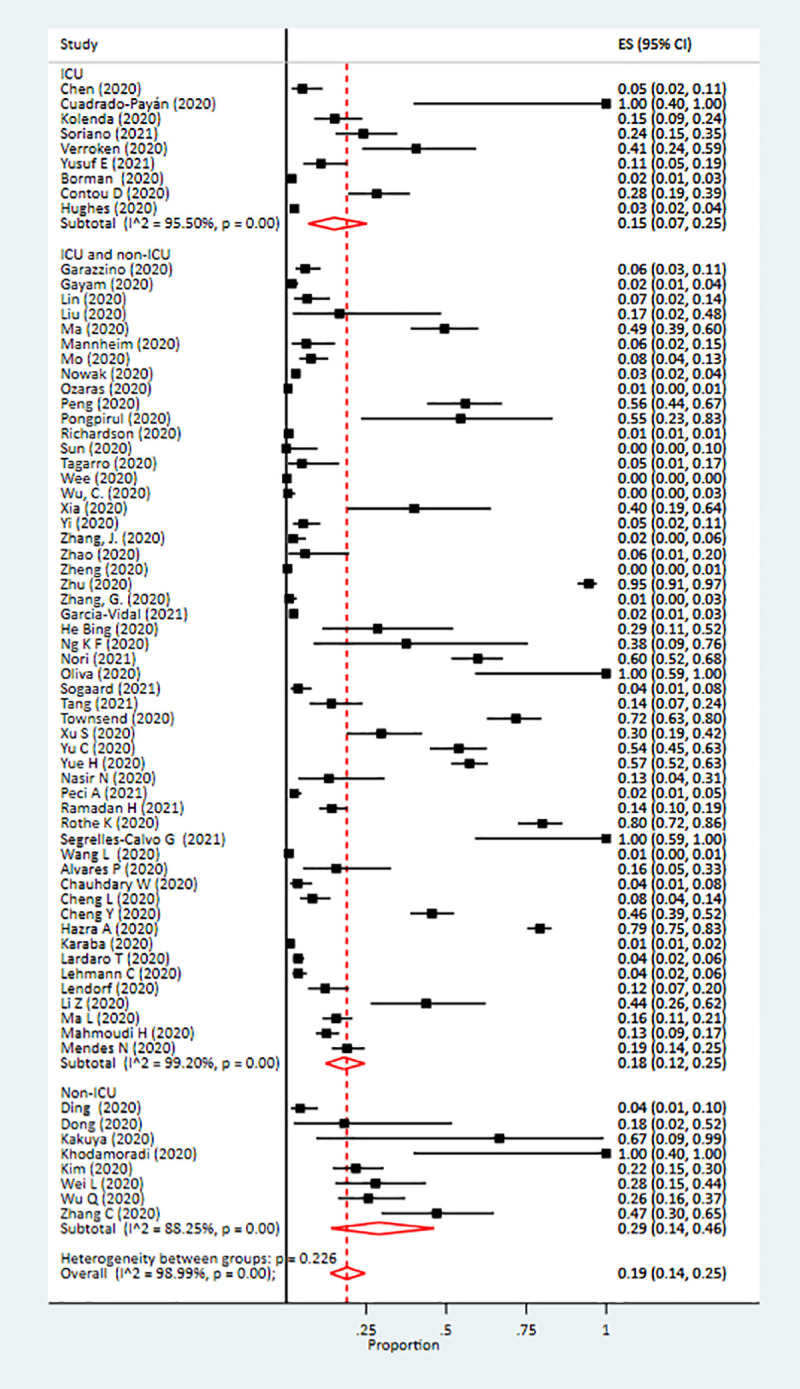
Forest plot of pooled prevalence of co-infection in patients infected with SARS-CoV-2.

**Fig 3:**
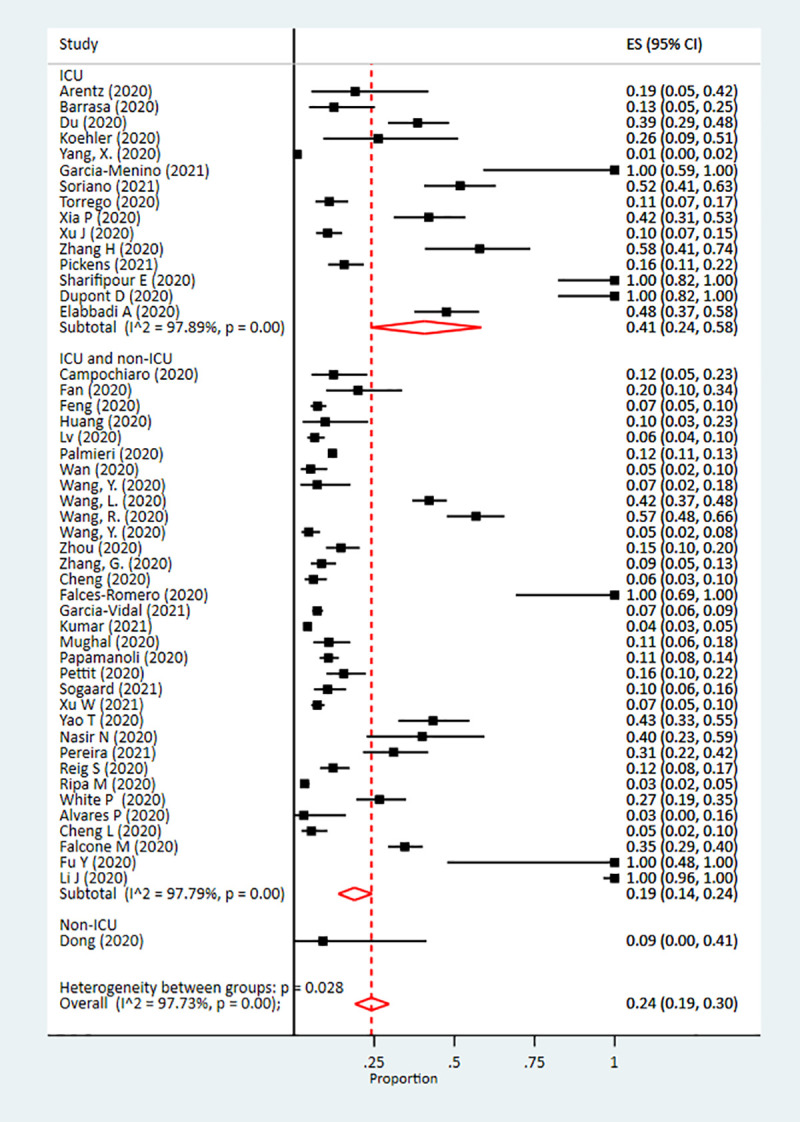
Forest plot of pooled prevalence of superinfection in patients infected with SARS-CoV-2.

The pooled prevalence of co-infection was 19% (95% confidence interval [CI]: 14%-25%; I^2^ = 98%). The highest prevalence of co-infection was observed among non-ICU patients at 29% (95% CI: 14%-46%), while it was 18% (95% CI: 12%-25%) among combined ICU and non-ICU patients, and 16% (95% CI: 8%-25%) among only ICU co-infected patients (Fig 2). The pooled prevalence of superinfection was 24% (95% CI: 19%-30%), with the highest prevalence among ICU patients (41% [95% CI: 24%-58%]) (Fig 3).

Pooled prevalence of pathogen type stratified by co- or superinfection was: viral co-infections, 10% (95% CI: 6%-14%) and viral superinfections, 4% (95% CI: 0%-10%); bacterial co-infections, 8% (95% CI: 5%-11%) and bacterial superinfections, 20% (95% CI: 13%-28%); and fungal co-infections, 4% (95% CI: 2%-7%) and fungal superinfections, 8% (95% CI: 4%-13%) (S1–S3 Figs).

Seventy-eight studies reported data on specific organisms associated with co-infection or superinfection in COVID-19 patients (Table 2). Among patients with co-infections, the three most frequently identified bacteria were *Klebsiella pneumoniae* (9.9%), *Streptococcus pneumoniae* (8.2%), and *Staphylococcus aureus* (7.7%). The three most frequently identified viruses among co-infected patients were influenza type A (22.3%), influenza type B (3.8%), and respiratory syncytial virus (3.8%). For fungi, *Aspergillus* was the most frequently reported among those co-infected.

**Table 2:** All identified organisms as a proportion of total number of organisms per pathogen.

Pathogen type	Co-infection (N = 1910) No. (%)	Superinfection (N = 480) No. (%)
Bacteria		
*Staphylococcus aureus*	148 (7.7)	13 (2.7)
*Haemophilus influenza*	127 (6.6)	6 (1.3)
*Mycoplasma pneumoniae*	82 (4.3)	6 (1.3)
*Acinetobacter spps*	78 (4.1)	107 (22.3)
*Escherichia coli*	73 (3.8)	33 (6.9)
*Stenotrophomonas maltophilia*	10 (0.5)	18 (3.8)
*Klebsiella pneumoniae*	189 (9.9)	28 (5.8)
*Streptococcus pneumoniae*	156 (8.2)	4 (0.8)
*Chlamydia pneumoniae*	29 (1.5)	0 (0)
*Bordetella*	3 (0.2)	0 (0)
*Moraxella catarrhalis*	32 (1.7)	2 (0.4)
*Pseudomonas*	67 (3.5)	52 (10.8)
*Enterococcus faecium*	14 (0.7)	22 (4.6)
**Viruses**		
Non-SARS-CoV-2^a^ coronavirus strains	38 (2.0)	9 (1.9)
Human influenza A	426 (22.3)	0 (0)
Human influenza *B*	73 (3.8)	0 (0)
Respiratory syncytial virus	72 (3.8)	2 (0.4)
Parainfluenza	17 (0.9)	0 (0)
Human metapneumovirus	20 (1.0)	9 (1.9)
Rhinovirus	68 (3.6)	11 (2.3)
Adenovirus	35 (1.8)	2 (0.4)
**Fungi**		
*Mucor*	6 (0.3)	1 (0.2)
*Candida spp*.	19 (1.0)	90 (18.8)
*Aspergillus*	128 (6.7)	65 (13.5)

^a^SARS-CoV-2: severe acute respiratory syndrome coronavirus 2.

Among those with superinfections, the three most frequently identified bacteria were *Acinetobacter spp*. (22.0%), *Pseudomonas* (10.8%), and *Escherichia coli* (6.9%). For viruses, Rhinovirus was the most frequently identified among those with superinfections, and for fungi, *Candida sp*. was the most frequent (18.8%).

The overall prevalence of comorbidities was 42% (95% CI: 35%-49%). Among those with co-infections, the prevalence of comorbidities was 32% (95% CI: 24%-41%), while it was 54% (95% CI: 42%-65%) among those who were super-infected.

Patients with a co-infection or superinfection had a higher odds of dying than those who only had SARS-CoV-2 infection (odds ratio [OR] = 3.31, 95% CI: 1.82–5.99). Subgroup analysis of mortality showed similar results, where the odds of death was higher among patients who were co-infected (OR = 2.84; 95% CI: 1.42–5.66) and those who were super-infected (OR = 3.54; 95% CI: 1.46–8.58). There was a higher prevalence of mechanical ventilation among patients with superinfections (45% [95% CI: 33%-58%]) compared to those with co-infections (10% [95% CI: 5%-16%]). Fifty studies reported data on average LOS. The average LOS for co-infected patients was 29 days (standard deviation [SD] = 6.7), while the average LOS for super-infected patients was 16 days (SD = 6.2). None of the studies included in this meta-analysis reported data on discharge disposition and readmissions.

### Risk of bias assessment

Sixty-two percent (73/118) of studies were rated as having low risk of bias, 34% (40/118) as having medium risk of bias, and 4% (5/118) as having a high risk of bias.

## Discussion

We found that 19% of patients with SARS-CoV-2 were co-infected with other pathogens, and the prevalence of co-infection was higher among patients who were not in the ICU (29%). We also found a higher prevalence of superinfection compared to co-infection (24%), particularly among ICU patients (41%). Further, we found that super-infected patients had a higher prevalence of mechanical ventilation and comorbidities, and a higher risk of death.

Two previous reviews found a prevalence of bacterial co-infection of 7–8% and viral co-infection of 3% in SARS-CoV-2 infected patients, which are lower than our estimates [11, 12]. We extended this work by distinguishing between super- and co-infection because of the different implications of co-infections vs. superinfections. In particular, bacteria and other pathogens have been shown to complicate viral pneumonia and lead to poor outcomes [137]. In addition, our review spanned a longer period of time and included many newer studies, which may further account for differences in prevalence data.

The three most frequently identified bacteria among co-infected patients in our study were *Klebsiella pneumonia*, *Streptococcus pneumoniae*, and *Staphylococcus aureus*. *Streptococcus pneumoniae* is a frequent cause of superinfection in other respiratory infections, such as influenza [138]. A study by Zhu et al. showed similar results [67], and a review by Lansbury et al. showed that *Klebsiella pneumoniae* and *Haemophilus influenza* were some of the most frequent bacterial co-infecting pathogens [11]. As expected, *Staphylococcus aureus* also was present in a sizeable number of cases. The most frequent bacteria identified in super-infected patients was *Acinetobacter spp*., which is a common infection, especially in ventilated patients [139].

In our study, the three most frequently identified viruses among co-infected patients were influenza type A, influenza type B, and respiratory syncytial virus. These findings are important particularly for influenza because testing constraints continue to exist, yet clinical presentation of influenza and SARS-CoV-2 is similar. There are major infection control and clinical implications of missing a SARS-CoV-2 or influenza diagnosis if co-infection is not considered and diagnostic testing for both pathogens is not undertaken.

Our findings have implications for infection preventionists, clinicians, and laboratory leaders. Respiratory virus diagnostic testing protocols should take into account that co-infection with SARS-CoV-2 is not infrequent, and therefore viral panel testing may be advisable in patients with compatible symptoms. Treatment protocols should also include assessment for co-infections, particularly influenza, so that appropriate treatment for both SARS-CoV-2 and influenza can be administered.

Another key finding from our study was that co-infection or superinfection was associated with an increased odds of death. This is consistent with other studies that have shown a positive association between co-infection or superinfection and increased risk of death among patients with the SARS-CoV-2 infection [140, 141].

Our study showed that antibiotics were administered in 98% of the 83 studies that reported this data. The type of antibiotics (i.e., broad or narrow spectrum) were not widely ascertainable, as these details were not provided in many studies. In the spirit of antibiotic stewardship, antibiotic use even in SARS-CoV-2 infected patients should be judicious and only in cases with an objective diagnosis of bacterial co-infection.

Our study has limitations. We were not able to assess important outcomes, such as discharge disposition and hospital readmissions, due to a lack of these data in the included studies. We were also not able to document time to superinfection, as the included studies did not report this information. Studies provided the number of patients with superinfections without stating the exact time when this determination was made after SARS-CoV-2 diagnosis. Most of the studies included in the meta-analysis were case series with their inherent limitations [142]. It is possible that some of the pathogens that were reported as superinfections or secondary infections were present but not tested for at admission and hence were co-infections. It was not possible to assess this from the studies. There was significant heterogeneity in the studies, as was anticipated given the variation in settings, patient populations, and diagnostic testing platforms across the studies.

## Conclusions

Our study showed that as many as 19% of patients with COVID-19 have co-infections and 24% have superinfections. The presence of either co-infection or superinfection was associated with poor outcomes, such as increased risk of mortality. Our findings support the need for diagnostic testing to identify and treat co-occurring respiratory infections among patients with SARS-CoV-2 infection.

## Supporting information

S1 Fig Forest plot of pooled prevalence of viral respiratory co-infections and viral superinfections in patients infected with SARS-CoV-2. (TIF)

S2 Fig Forest plot of pooled prevalence of bacterial co-infections and bacterial superinfections in patients infected with SARS-CoV-2. (TIF)

S3 Fig Forest plot of pooled prevalence of fungal co-infections and fungal superinfections in patients infected with SARS-CoV-2. (TIF)

S1 File Study protocol. (PDF)

S2 File Supplementary material: Search strategies, COVID-19 and co-infections, and final search. (PDF)

S3 File PRISMA 2009 checklist. (PDF)

S4 File Data used for the analysis. (XLSX)

## References

[1] Centers for Disease Control and Prevention. Coronavirus Disease 2019 (COVID-19): cases in US 2020 [Available from: https://www.cdc.gov/coronavirus/2019-ncov/cases-updates/cases-in-us.html.

[2] The World Health Organization. Coronavirus disease (COVID-19) Pandemic 2020 [Available from: https://www.who.int/emergencies/diseases/novel-coronavirus-2019.

[3] The World Health Organization. Modes of transmission of virus causing COVID-19: implications for IPC precaution recommendations 2019 [Available from: https://www.who.int/news-room/commentaries/detail/modes-of-transmission-of-virus-causing-covid-19-implications-for-ipc-precaution-recommendations.

[4] Wang Y, Chen Y, Qin Q. Unique epidemiological and clinical features of the emerging 2019 novel coronavirus pneumonia (COVID-19) implicate special control measures. J Med Virol. 2020. 10.1002/jmv.25748 32134116 PMC7228347

[5] Arons MM, Hatfield KM, Reddy SC, Kimball A, James A, Jacobs JR, et al. Presymptomatic SARS-CoV-2 Infections and Transmission in a Skilled Nursing Facility. N Engl J Med. 2020. 10.1056/NEJMoa2008457 PMC7200056 32329971

[6] Chertow DS, Memoli MJ. Bacterial coinfection in influenza: a grand rounds review. JAMA. 2013;309(3):275–82. 10.1001/jama.2012.194139 23321766

[7] Morens DM, Taubenberger JK, Fauci AS. Predominant role of bacterial pneumonia as a cause of death in pandemic influenza: implications for pandemic influenza preparedness. J Infect Dis. 2008;198(7):962–70. 10.1086/591708 18710327 PMC2599911

[8] Lin D, Liu L, Zhang M, Hu Y, Yang Q, Guo J, et al. Co-infections of SARS-CoV-2 with multiple common respiratory pathogens in infected patients. Sci China Life Sci. 2020;63(4):606–9. 10.1007/s11427-020-1668-5 32170625 PMC7089461

[9] Nowak MD, Sordillo EM, Gitman MR, Paniz Mondolfi AE. Co-infection in SARS-CoV-2 infected Patients: Where Are Influenza Virus and Rhinovirus/Enterovirus? Journal of Medical Virology. 2020. 10.1002/jmv.25953 32352574 PMC7267652

[10] Wang M, Wu Q, Xu W, Qiao B, Wang J, Zheng H, et al. Clinical diagnosis of 8274 samples with 2019-novel coronavirus in Wuhan. medRxiv. 2020:2020.02.12.20022327.

[11] Lansbury L, Lim B, Baskaran V, Lim WS. Co-infections in people with COVID-19: a systematic review and meta-analysis. The Journal of infection. 2020. 10.1016/j.jinf.2020.05.046 32473235 PMC7255350

[12] Rawson TM, Moore LSP, Zhu N, Ranganathan N, Skolimowska K, Gilchrist M, et al. Bacterial and Fungal Coinfection in Individuals With Coronavirus: A Rapid Review To Support COVID-19 Antimicrobial Prescribing. Clinical Infectious Diseases. 2020. 10.1093/cid/ciaa530 32358954 PMC7197596

[13] Garcia-Vidal C, Sanjuan G, Moreno-García E, Puerta-Alcalde P, Garcia-Pouton N, Chumbita M, et al. Incidence of co-infections and superinfections in hospitalized patients with COVID-19: a retrospective cohort study. Clinical Microbiology and Infection. 10.1016/j.cmi.2020.07.041 32745596 PMC7836762

[14] Moher D, Shamseer L, Clarke M, Ghersi D, Liberati A, Petticrew M, et al. Preferred reporting items for systematic review and meta-analysis protocols (PRISMA-P) 2015 statement. Systematic reviews. 2015;4:1. 10.1186/2046-4053-4-1 25554246 PMC4320440

[15] Musuuza J, Watson L, Parmasad V, Putman-Buehler N, Christensen L, Safdar N. The prevalence and outcomes of co-infection with COVID-19 and other pathogens: a rapid systematic review and meta-analysis. PROSPERO 2020 CRD42020189763 [Available from: https://www.crd.york.ac.uk/prospero/display_record.php?ID=CRD42020189763. 10.1371/journal.pone.0251170 PMC8101968 33956882

[16] Murad MH, Sultan S, Haffar S, Bazerbachi F. Methodological quality and synthesis of case series and case reports. BMJ Evidence-Based Medicine. 2018;23(2):60. 10.1136/bmjebm-2017-110853 29420178 PMC6234235

[17] Downs SH, Black N. The feasibility of creating a checklist for the assessment of the methodological quality both of randomised and non-randomised studies of health care interventions. J Epidemiol Community Health. 1998;52(6):377–84. 10.1136/jech.52.6.377 9764259 PMC1756728

[18] Nyaga VN, Arbyn M, Aerts M. Metaprop: a Stata command to perform meta-analysis of binomial data. Arch Public Health. 2014;72(1):39. 10.1186/2049-3258-72-39 25810908 PMC4373114

[19] DerSimonian R, Laird N. Meta-analysis in clinical trials. Control Clin Trials. 1986;7(3):177–88. 10.1016/0197-2456(86)90046-2 3802833

[20] Hunter JP, Saratzis A, Sutton AJ, Boucher RH, Sayers RD, Bown MJ. In meta-analyses of proportion studies, funnel plots were found to be an inaccurate method of assessing publication bias. J Clin Epidemiol. 2014;67(8):897–903. 10.1016/j.jclinepi.2014.03.003 24794697

[21] Lin L. Graphical augmentations to sample-size-based funnel plot in meta-analysis. Res Synth Methods. 2019;10(3):376–88. 10.1002/jrsm.1340 30664834 PMC6642847

[22] Arentz M, Yim E, Klaff L, Lokhandwala S, Riedo FX, Chong M, et al. Characteristics and Outcomes of 21 Critically Ill Patients With COVID-19 in Washington State. JAMA. 2020;323(16):1612–4. 10.1001/jama.2020.4326 32191259 PMC7082763

[23] Barrasa H, Rello J, Tejada S, Martín A, Balziskueta G, Vinuesa C, et al. SARS-CoV-2 in Spanish Intensive Care Units: Early experience with 15-day survival in Vitoria. Anaesth Crit Care Pain Med. 2020. 10.1016/j.accpm.2020.04.001 32278670 PMC7144603

[24] Campochiaro C, Della-Torre E, Cavalli G, De Luca G, Ripa M, Boffini N, et al. Efficacy and safety of tocilizumab in severe COVID-19 patients: a single-centre retrospective cohort study. European Journal of Internal Medicine. 2020;76:43–9. 10.1016/j.ejim.2020.05.021 32482597 PMC7242960

[25] Chen N, Zhou M, Dong X, Qu J, Gong F, Han Y, et al. Epidemiological and clinical characteristics of 99 cases of 2019 novel coronavirus pneumonia in Wuhan, China: a descriptive study. Lancet. 2020;395(10223):507–13. 10.1016/S0140-6736(20)30211-7 32007143 PMC7135076

[26] Cuadrado-Payán E, Montagud-Marrahi E, Torres-Elorza M, Bodro M, Blasco M, Poch E, et al. SARS-CoV-2 and influenza virus co-infection. The Lancet. 2020;395(10236):e84. 10.1016/S0140-6736(20)31052-7 32423586 PMC7200126

[27] Ding Q, Lu P, Fan Y, Xia Y, Liu M. The clinical characteristics of pneumonia patients coinfected with 2019 novel coronavirus and influenza virus in Wuhan, China. J Med Virol. 2020. 10.1002/jmv.25781 32196707 PMC7228290

[28] Dong X, Cao YY, Lu XX, Zhang JJ, Du H, Yan YQ, et al. Eleven faces of coronavirus disease 2019. Allergy. 2020. 10.1111/all.14289 32196678 PMC7228397

[29] Du RH, Liu LM, Yin W, Wang W, Guan LL, Yuan ML, et al. Hospitalization and Critical Care of 109 Decedents with COVID-19 Pneumonia in Wuhan, China. Ann Am Thorac Soc. 2020. 10.1513/AnnalsATS.202003-225OC 32255382 PMC7328178

[30] Fan W, Yumin Z, Zhongfang W, Min X, Zhe S, Zhiqiang T, et al. Clinical characteristics of COVID-19 infection in chronic obstructive pulmonary disease: a multicenter, retrospective, observational study. Journal of Thoracic Disease. 2020;12(5):1811–23. 10.21037/jtd-20-1914 32642086 PMC7330323

[31] Feng Y, Ling Y, Bai T, Xie Y, Huang J, Li J, et al. COVID-19 with Different Severities: A Multicenter Study of Clinical Features. American Journal of Respiratory and Critical Care Medicine. 2020;201(11):1380–8. 10.1164/rccm.202002-0445OC 32275452 PMC7258639

[32] Garazzino S, Montagnani C, Donà D, Meini A, Felici E, Vergine G, et al. Multicentre Italian study of SARS-CoV-2 infection in children and adolescents, preliminary data as at 10 April 2020. Euro surveillance: bulletin Europeen sur les maladies transmissibles = European communicable disease bulletin. 2020;25(18). 10.2807/1560-7917.ES.2020.25.18.2000600 32400362 PMC7219028

[33] Gayam V, Konala VM, Naramala S, Garlapati PR, Merghani MA, Regmi N, et al. Presenting characteristics, comorbidities, and outcomes of patients coinfected with COVID-19 and Mycoplasma pneumoniae in the USA. Journal of Medical Virology. 2020. 10.1002/jmv.26026 PMC7280653 32449972

[34] Huang C, Wang Y, Li X, Ren L, Zhao J, Hu Y, et al. Clinical features of patients infected with 2019 novel coronavirus in Wuhan, China. Lancet. 2020;395(10223):497–506. 10.1016/S0140-6736(20)30183-5 31986264 PMC7159299

[35] Kakuya F, Okubo H, Fujiyasu H, Wakabayashi I, Syouji M, Kinebuchi T. The first pediatric patients with coronavirus disease 2019 (COVID-19) in Japan; The risk of co-infection with other respiratory viruses. Japanese journal of infectious diseases. 2020. 10.7883/yoken.JJID.2020.181 32475878

[36] Khodamoradi Z, Moghadami M, Lotfi M. Co-infection of coronavirus disease 2019 and influenza a: A report from Iran. Archives of Iranian Medicine. 2020;23(4):239–43. 10.34172/aim.2020.04 32271596

[37] Kim D, Quinn J, Pinsky B, Shah NH, Brown I. Rates of Co-infection Between SARS-CoV-2 and Other Respiratory Pathogens. JAMA. 2020;323(20):2085–6. 10.1001/jama.2020.6266 32293646 PMC7160748

[38] Koehler P, Cornely OA, Böttiger BW, Dusse F, Eichenauer DA, Fuchs F, et al. COVID-19 associated pulmonary aspergillosis. Mycoses. 2020;63(6):528–34. 10.1111/myc.13096 32339350 PMC7267243

[39] Lian J, Jin X, Hao S, Cai H, Zhang S, Zheng L, et al. Analysis of Epidemiological and Clinical features in older patients with Corona Virus Disease 2019 (COVID-19) out of Wuhan. Clinical Infectious Diseases. 2020. 10.1093/cid/ciaa242 PMC7184356 32211844

[40] Liu Y, Yang Y, Zhang C, Huang F, Wang F, Yuan J, et al. Clinical and biochemical indexes from 2019-nCoV infected patients linked to viral loads and lung injury. Sci China Life Sci. 2020;63(3):364–74. 10.1007/s11427-020-1643-8 32048163 PMC7088566

[41] Lv Z, Cheng S, Le J, Huang J, Feng L, Zhang B, et al. Clinical characteristics and co-infections of 354 hospitalized patients with COVID-19 in Wuhan, China: a retrospective cohort study. Microbes Infect. 2020. 10.1016/j.micinf.2020.05.007 32425649 PMC7233257

[42] Ma S, Lai X, Chen Z, Tu S, Qin K. Clinical Characteristics of Critically Ill Patients Co-infected with SARS-CoV-2 and the Influenza Virus in Wuhan, China. International Journal of Infectious Diseases. 2020. 10.1016/j.ijid.2020.05.068 32470606 PMC7250072

[43] Mannheim J, Gretsch S, Layden JE, Fricchione MJ. Characteristics of Hospitalized Pediatric COVID-19 Cases—Chicago, Illinois, March—April 2020. J Pediatric Infect Dis Soc. 2020. 10.1093/jpids/piaa070 PMC7542980 32479632

[44] Mo P, Xing Y, Xiao Y, Deng L, Zhao Q, Wang H, et al. Clinical characteristics of refractory COVID-19 pneumonia in Wuhan, China. Clinical Infectious Diseases. 2020. 10.1093/cid/ciaa270 32173725 PMC7184444

[45] Ozaras R, Cirpin R, Duran A, Duman H, Arslan O, Bakcan Y, et al. Influenza and COVID-19 Co-infection: Report of 6 cases and review of the Literature. Journal of Medical Virology. 2020. 10.1002/jmv.26125 32497283

[46] Palmieri L, Vanacore N, Donfrancesco C, Lo Noce C, Canevelli M, Punzo O, et al. Clinical Characteristics of Hospitalized Individuals Dying with COVID-19 by Age Group in Italy. Journals of Gerontology Series A: Biological Sciences and Medical Sciences. 2020. 10.1093/gerona/glaa140 32506122 PMC7314182

[47] Peng H, Gao P, Xu Q, Liu M, Peng J, Wang Y, et al. Coronavirus disease 2019 in children: Characteristics, antimicrobial treatment, and outcomes. Journal of Clinical Virology. 2020;128. 10.1016/j.jcv.2020.104425 PMC7204737 32446167

[48] Pongpirul WA, Mott JA, Woodring JV, Uyeki TM, MacArthur JR, Vachiraphan A, et al. Clinical Characteristics of Patients Hospitalized with Coronavirus Disease, Thailand. Emerging Infectious Diseases. 2020;26(7). 10.3201/eid2607.200598 PMC7323520 32267826

[49] Richardson S, Hirsch JS, Narasimhan M, Crawford JM, McGinn T, Davidson KW, et al. Presenting Characteristics, Comorbidities, and Outcomes Among 5700 Patients Hospitalized With COVID-19 in the New York City Area. Jama. 2020;323(20):2052–9. 10.1001/jama.2020.6775 32320003 PMC7177629

[50] Sun D, Chen X, Li H, Lu XX, Xiao H, Zhang FR, et al. SARS-CoV-2 infection in infants under 1 year of age in Wuhan City, China. World Journal of Pediatrics. 2020:1–7. 10.1007/s12519-020-00368-y 32504360 PMC7274073

[51] Tagarro A, Epalza C, Santos M, Sanz-Santaeufemia FJ, Otheo E, Moraleda C, et al. Screening and Severity of Coronavirus Disease 2019 (COVID-19) in Children in Madrid, Spain. JAMA Pediatr. 2020. 10.1001/jamapediatrics.2020.1346 32267485 PMC7142799

[52] Wan S, Xiang Y, Fang W, Zheng Y, Li B, Hu Y, et al. Clinical features and treatment of COVID-19 patients in northeast Chongqing. Journal of Medical Virology. 2020;92(7):797–806. 10.1002/jmv.25783 32198776 PMC7228368

[53] Wang Y, Liu Y, Liu L, Wang X, Luo N, Li L. Clinical Outcomes in 55 Patients With Severe Acute Respiratory Syndrome Coronavirus 2 Who Were Asymptomatic at Hospital Admission in Shenzhen, China. Journal of Infectious Diseases. 2020;221(11):1770–4. 10.1093/infdis/jiaa119 32179910 PMC7184401

[54] Wang L, He W, Yu X, Hu D, Bao M, Liu H, et al. Coronavirus disease 2019 in elderly patients: Characteristics and prognostic factors based on 4-week follow-up. J Infect. 2020;80(6):639–45. 32240670 10.1016/j.jinf.2020.03.019 PMC7118526

[55] Wang R, Pan M, Zhang X, Han M, Fan X, Zhao F, et al. Epidemiological and clinical features of 125 Hospitalized Patients with COVID-19 in Fuyang, Anhui, China. International Journal of Infectious Diseases. 2020;95:421–8. 10.1016/j.ijid.2020.03.070 32289565 PMC7151431

[56] Wang Y, Zhang D, Du G, Du R, Zhao J, Jin Y, et al. Remdesivir in adults with severe COVID-19: a randomised, double-blind, placebo-controlled, multicentre trial. Lancet. 2020;395(10236):1569–78. 10.1016/S0140-6736(20)31022-9 32423584 PMC7190303

[57] Wee LE, Ko KKK, Ho WQ, Kwek GTC, Tan TT, Wijaya L. Community-acquired viral respiratory infections amongst hospitalized inpatients during a COVID-19 outbreak in Singapore: co-infection and clinical outcomes. Journal of clinical virology: the official publication of the Pan American Society for Clinical Virology. 2020;128:104436. 10.1016/j.jcv.2020.104436 32447256 PMC7235565

[58] Wu C, Chen X, Cai Y, Xia J, Zhou X, Xu S, et al. Risk Factors Associated With Acute Respiratory Distress Syndrome and Death in Patients With Coronavirus Disease 2019 Pneumonia in Wuhan, China. JAMA Intern Med. 2020. 10.1001/jamainternmed.2020.0994 32167524 PMC7070509

[59] Xia W, Shao J, Guo Y, Peng X, Li Z, Hu D. Clinical and CT features in pediatric patients with COVID-19 infection: Different points from adults. Pediatric Pulmonology. 2020;55(5):1169–74. 10.1002/ppul.24718 32134205 PMC7168071

[60] Yang X, Yu Y, Xu J, Shu H, Xia J, Liu H, et al. Clinical course and outcomes of critically ill patients with SARS-CoV-2 pneumonia in Wuhan, China: a single-centered, retrospective, observational study. Lancet Respir Med. 2020;8(5):475–81. 10.1016/S2213-2600(20)30079-5 32105632 PMC7102538

[61] Yi SG, Rogers AW, Saharia A, Aoun M, Faour R, Abdelrahim M, et al. Early Experience With COVID-19 and Solid Organ Transplantation at a US High-volume Transplant Center. Transplantation. 2020. 10.1097/TP.0000000000003339 PMC7302089 32496357

[62] Zhang JJ, Dong X, Cao YY, Yuan YD, Yang YB, Yan YQ, et al. Clinical characteristics of 140 patients infected with SARS-CoV-2 in Wuhan, China. Allergy. 2020. 10.1111/all.14238 32077115

[63] Zhang G, Hu C, Luo L, Fang F, Chen Y, Li J, et al. Clinical features and short-term outcomes of 221 patients with COVID-19 in Wuhan, China. Journal of clinical virology: the official publication of the Pan American Society for Clinical Virology. 2020;127:104364. 10.1016/j.jcv.2020.104364 32311650 PMC7194884

[64] Zhao D, Yao F, Wang L, Zheng L, Gao Y, Ye J, et al. A comparative study on the clinical features of COVID-19 pneumonia to other pneumonias. Clinical Infectious Diseases. 2020. 10.1093/cid/ciaa247 32161968 PMC7108162

[65] Zheng X, Wang H, Su Z, Li W, Yang D, Deng F, et al. Co-infection of SARS-CoV-2 and Influenza virus in Early Stage of the COVID-19 Epidemic in Wuhan, China. Journal of Infection. 2020. 10.1016/j.jinf.2020.05.041 32474045 PMC7255712

[66] Zhou F, Yu T, Du R, Fan G, Liu Y, Liu Z, et al. Clinical course and risk factors for mortality of adult inpatients with COVID-19 in Wuhan, China: a retrospective cohort study. Lancet. 2020;395(10229):1054–62. 10.1016/S0140-6736(20)30566-3 32171076 PMC7270627

[67] Zhu X, Ge Y, Wu T, Zhao K, Chen Y, Wu B, et al. Co-infection with respiratory pathogens among COVID-2019 cases. Virus Research. 2020;285. 10.1016/j.virusres.2020.198005 32408156 PMC7213959

[68] Alvares PA. SARS-COV-2 AND RESPIRATORY SYNCYTIAL VIRUS COINFECTION IN HOSPITALIZED PEDIATRIC PATIENTS. Pediatric Infectious Disease Journal. 2021. 10.1097/INF.0000000000003057 33464015

[69] Borman AM, Palmer MD, Fraser M, Patterson Z, Mann C, Oliver D, et al. COVID-19-Associated Invasive Aspergillosis: Data from the UK National Mycology Reference Laboratory. Journal of Clinical Microbiology. 2020;59(1). 10.1128/JCM.02136-20 33087440 PMC7771443

[70] Chauhdary WA, Chong PL, Mani BI, Asli R, Momin RN, Abdullah MS, et al. Primary Respiratory Bacterial Coinfections in Patients with COVID-19. The American journal of tropical medicine and hygiene. 2020;103(2):917–9. 10.4269/ajtmh.20-0498 32500854 PMC7410441

[71] Cheng LS, Chau SK, Tso EY, Tsang SW, Li IY, Wong BK, et al. Bacterial co-infections and antibiotic prescribing practice in adults with COVID-19: experience from a single hospital cluster. Ther Adv Infect Dis. 2020;7:2049936120978095. 10.1177/2049936120978095 33335724 PMC7724262

[72] Cheng Y, Ma J, Wang H, Wang X, Hu Z, Li H, et al. Co-infection of influenza A virus and SARS-CoV-2: A retrospective cohort study. Journal of Medical Virology. 2021. 10.1002/jmv.26817 33475159 PMC8013771

[73] Cheng K, He M, Shu Q, Wu M, Chen C, Xue Y. Analysis of the Risk Factors for Nosocomial Bacterial Infection in Patients with COVID-19 in a Tertiary Hospital. Risk Management and Healthcare Policy. 2020;13:2593–9. 10.2147/RMHP.S277963 33223859 PMC7671853

[74] Contou D, Claudinon A, Pajot O, Micaelo M, Longuet Flandre P, Dubert M, et al. Bacterial and viral co-infections in patients with severe SARS-CoV-2 pneumonia admitted to a French ICU. Annals of intensive care. 2020;10(1):119. 10.1186/s13613-020-00736-x 32894364 PMC7475952

[75] Dupont D, Menotti J, Turc J, Miossec C, Wallet F, Richard JC, et al. Pulmonary aspergillosis in critically ill patients with Coronavirus Disease 2019 (COVID-19). Medical Mycology. 2021;59(1):110–4. 10.1093/mmy/myaa078 32914189 PMC7499748

[76] Elabbadi A, Turpin M, Gerotziafas GT, Teulier M, Voiriot G, Fartoukh M. Bacterial coinfection in critically ill COVID-19 patients with severe pneumonia. Infection. 2021. 10.1007/s15010-020-01553-x 33393065 PMC7779094

[77] Falces-Romero I, Ruiz-Bastián M, Díaz-Pollán B, Maseda E, García-Rodríguez J. Isolation of Aspergillus spp. in respiratory samples of patients with COVID-19 in a Spanish Tertiary Care Hospital. Mycoses. 2020. 10.1111/myc.13155 PMC7436624 32749040

[78] Falcone M, Tiseo G, Giordano C, Leonildi A, Menichini M, Vecchione A, et al. Predictors of hospital-acquired bacterial and fungal superinfections in COVID-19: a prospective observational study. Journal of Antimicrobial Chemotherapy. 2020. 10.1093/jac/dkaa530 PMC7799007 33374002

[79] Fu Y, Yang Q, Xu M, Kong H, Chen H, Fu Y, et al. Secondary Bacterial Infections in Critical Ill Patients With Coronavirus Disease 2019. Open Forum Infect Dis. 2020;7(6):ofaa220. 10.1093/ofid/ofaa220 32613024 PMC7313762

[80] Garcia-Menino I, Forcelledo L, Rosete Y, Garcia-Prieto E, Escudero D, Fernandez J. Spread of OXA-48-producing Klebsiella pneumoniae among COVID-19-infected patients: The storm after the storm. Journal of infection and public health. 2021;14(1):50–2. 10.1016/j.jiph.2020.11.001 33341484 PMC7713590

[81] Garcia-Vidal C, Sanjuan G, Moreno-Garcia E, Puerta-Alcalde P, Garcia-Pouton N, Chumbita M, et al. Incidence of co-infections and superinfections in hospitalized patients with COVID-19: a retrospective cohort study. Clin Microbiol Infect. 2021;27(1):83–8. 10.1016/j.cmi.2020.07.041 32745596 PMC7836762

[82] Gouzien L, Cocherie T, Eloy O, Legriel S, Bedos JP, Simon C, et al. Invasive Aspergillosis Associated with severe COVID-19: A Word of Caution. Infect Dis Now. 2021. 10.1016/j.idnow.2020.12.008 33490993 PMC7813486

[83] Hashemi SA, Safamanesh S, Ghasemzadeh-Moghaddam H, Ghafouri M, Azimian A. High prevalence of SARS-CoV-2 and influenza A virus (H1N1) coinfection in dead patients in Northeastern Iran. Journal of Medical Virology. 2021;93(2):1008–12. 10.1002/jmv.26364 32720703

[84] Hazra A, Collison M, Pisano J, Kumar M, Oehler C, Ridgway JP. Coinfections with SARS-CoV-2 and other respiratory pathogens. Infect Control Hosp Epidemiol. 2020;41(10):1228–9. 10.1017/ice.2020.322 32616098 PMC7360954

[85] He B, Wang J, Wang Y, Zhao J, Huang J, Tian Y, et al. The Metabolic Changes and Immune Profiles in Patients With COVID-19. Frontiers in Immunology. 2020;11:2075. 10.3389/fimmu.2020.02075 32983157 PMC7485144

[86] Hirotsu Y, Maejima M, Shibusawa M, Amemiya K, Nagakubo Y, Hosaka K, et al. Analysis of Covid-19 and non-Covid-19 viruses, including influenza viruses, to determine the influence of intensive preventive measures in Japan. Journal of Clinical Virology. 2020;129:104543. 10.1016/j.jcv.2020.104543 32663787 PMC7340051

[87] Hughes S, Troise O, Donaldson H, Mughal N, Moore LSP. Bacterial and fungal coinfection among hospitalized patients with COVID-19: a retrospective cohort study in a UK secondary-care setting. Clinical Microbiology and Infection. 2020;26(10):1395–9. 10.1016/j.cmi.2020.06.025 32603803 PMC7320692

[88] Karaba SM, Jones G, Helsel T, Smith LL, Avery R, Dzintars K, et al. Prevalence of Co-infection at the Time of Hospital Admission in COVID-19 Patients, A Multicenter Study. Open Forum Infect Dis. 2021;8(1):ofaa578. 10.1093/ofid/ofaa578 33447639 PMC7793465

[89] Kolenda C, Ranc AG, Boisset S, Caspar Y, Carricajo A, Souche A, et al. Assessment of Respiratory Bacterial Coinfections Among Severe Acute Respiratory Syndrome Coronavirus 2-Positive Patients Hospitalized in Intensive Care Units Using Conventional Culture and BioFire, FilmArray Pneumonia Panel Plus Assay. Open Forum Infect Dis. 2020;7(11):ofaa484. 10.1093/ofid/ofaa484 33204762 PMC7654374

[90] Kumar G, Adams A, Hererra M, Rojas ER, Singh V, Sakhuja A, et al. Predictors and outcomes of healthcare-associated infections in COVID-19 patients. International journal of infectious diseases: IJID: official publication of the International Society for Infectious Diseases. 2021;104:287–92. 10.1016/j.ijid.2020.11.135 33207271 PMC7666872

[91] Lardaro T, Wang AZ, Bucca A, Croft A, Glober N, Holt DB, et al. Characteristics of COVID-19 Patients with Bacterial Co-infection Admitted to the Hospital from the Emergency Department in a Large Regional Healthcare System. Journal of Medical Virology. 2021. 10.1002/jmv.26795 PMC8014736 33448423

[92] Lehmann CJ, Pho MT, Pitrak D, Ridgway JP, Pettit NN. Community Acquired Co-infection in COVID-19: A Retrospective Observational Experience. Clinical Infectious Diseases. 2020. 10.1093/cid/ciaa902 PMC7337635 32604413

[93] Lendorf ME, Boisen MK, Kristensen PL, Lokkegaard ECL, Krog SM, Brandi L, et al. Characteristics and early outcomes of patients hospitalised for COVID-19 in North Zealand, Denmark. Dan Med J. 2020;67(9). 32800073

[94] Li J, Wang J, Yang Y, Cai P, Cao J, Cai X, et al. Etiology and antimicrobial resistance of secondary bacterial infections in patients hospitalized with COVID-19 in Wuhan, China: a retrospective analysis. Antimicrobial resistance and infection control. 2020;9(1):153. 10.1186/s13756-020-00819-1 32962731 PMC7506844

[95] Li Z, Chen ZM, Chen LD, Zhan YQ, Li SQ, Cheng J, et al. Coinfection with SARS-CoV-2 and other respiratory pathogens in patients with COVID-19 in Guangzhou, China. J Med Virol. 2020;92(11):2381–3. 10.1002/jmv.26073 32462695 PMC7283743

[96] Ma L, Wang W, Le Grange JM, Wang X, Du S, Li C, et al. Coinfection of SARS-CoV-2 and Other Respiratory Pathogens. Infect Drug Resist. 2020;13:3045–53. 10.2147/IDR.S267238 32922049 PMC7457866

[97] Mahmoudi H. Bacterial co-infections and antibiotic resistance in patients with COVID-19. GMS hygiene and infection control. 2020;15:Doc35. 10.3205/dgkh000370 33391970 PMC7747008

[98] Goncalves Mendes Neto A, Lo KB, Wattoo A, Salacup G, Pelayo J, DeJoy R, 3rd, et al. Bacterial infections and patterns of antibiotic use in patients with COVID-19. J Med Virol. 2021;93(3):1489–95. 10.1002/jmv.26441 32808695 PMC7461450

[99] Mughal MS, Kaur IP, Jaffery AR, Dalmacion DL, Wang C, Koyoda S, et al. COVID-19 patients in a tertiary US hospital: Assessment of clinical course and predictors of the disease severity. Respir Med. 2020;172:106130. 10.1016/j.rmed.2020.106130 32896798 PMC7455149

[100] Nasir N, Mahmood F, Habib K, Khanum I, Jamil B. Tocilizumab for COVID-19 Acute Respiratory Distress Syndrome: Outcomes Assessment Using the WHO Ordinal Scale. Cureus. 2020;12(12):e12290. 10.7759/cureus.12290 33510989 PMC7829612

[101] Nasir N, Farooqi J, Mahmood SF, Jabeen K. COVID-19-associated pulmonary aspergillosis (CAPA) in patients admitted with severe COVID-19 pneumonia: An observational study from Pakistan. Mycoses. 2020;63(8):766–70. 10.1111/myc.13135 32585069 PMC7361517

[102] Ng KF, Bandi S, Bird PW, Wei-Tze Tang J. COVID-19 in Neonates and Infants: Progression and Recovery. Pediatric Infectious Disease Journal. 2020;39(7):e140–e2. 10.1097/INF.0000000000002738 32384398

[103] Nori P, Cowman K, Chen V, Bartash R, Szymczak W, Madaline T, et al. Bacterial and fungal coinfections in COVID-19 patients hospitalized during the New York City pandemic surge. Infection Control and Hospital Epidemiology. 2021;42(1):84–8. 10.1017/ice.2020.368 32703320 PMC7417979

[104] Obata R, Maeda T, Do DR, Kuno T. Increased secondary infection in COVID-19 patients treated with steroids in New York City. Japanese journal of infectious diseases. 2020. 10.7883/yoken.JJID.2020.884 33390434

[105] Oliva A, Siccardi G, Migliarini A, Cancelli F, Carnevalini M, D’Andria M, et al. Co-infection of SARS-CoV-2 with Chlamydia or Mycoplasma pneumoniae: a case series and review of the literature. Infection. 2020;48(6):871–7. 10.1007/s15010-020-01483-8 32725598 PMC7386385

[106] Papamanoli A, Yoo J, Grewal P, Predun W, Hotelling J, Jacob R, et al. High-dose methylprednisolone in nonintubated patients with severe COVID-19 pneumonia. European journal of clinical investigation. 2021;51(2):e13458. 10.1111/eci.13458 33219551 PMC7744876

[107] Peci A, Tran V, Guthrie JL, Li Y, Nelson P, Schwartz KL, et al. Prevalence of Co-Infections with Respiratory Viruses in Individuals Investigated for SARS-CoV-2 in Ontario, Canada. Viruses. 2021;13(1). 10.3390/v13010130 33477649 PMC7831481

[108] Pereira MR, Aversa MM, Farr MA, Miko BA, Aaron JG, Mohan S, et al. Tocilizumab for severe COVID-19 in solid organ transplant recipients: a matched cohort study. American Journal of Transplantation. 2020;20(11):3198–205. 10.1111/ajt.16314 32946668 PMC7537322

[109] Pettit NN, Nguyen CT, Mutlu GM, Wu D, Kimmig L, Pitrak D, et al. Late onset infectious complications and safety of tocilizumab in the management of COVID-19. J Med Virol. 2021;93(3):1459–64. 10.1002/jmv.26429 32790075 PMC7436682

[110] Pickens CO, Gao CA, Cuttica M, Smith SB, Pesce L, Grant R, et al. Bacterial superinfection pneumonia in SARS-CoV-2 respiratory failure. medRxiv. 2021. 10.1101/2021.01.12.20248588 33469593 PMC7814839

[111] Ramadan HK, Mahmoud MA, Aburahma MZ, Elkhawaga AA, El-Mokhtar MA, Sayed IM, et al. Predictors of Severity and Co-Infection Resistance Profile in COVID-19 Patients: First Report from Upper Egypt. Infect Drug Resist. 2020;13:3409–22. 10.2147/IDR.S272605 33116660 PMC7547142

[112] Rieg S, von Cube M, Kalbhenn J, Utzolino S, Pernice K, Bechet L, et al. COVID-19 in-hospital mortality and mode of death in a dynamic and non-restricted tertiary care model in Germany. PloS one. 2020;15(11):e0242127. 10.1371/journal.pone.0242127 33180830 PMC7660518

[113] Ripa M, Galli L, Poli A, Oltolini C, Spagnuolo V, Mastrangelo A, et al. Secondary infections in patients hospitalized with COVID-19: incidence and predictive factors. Clinical Microbiology and Infection. 2020. 10.1016/j.cmi.2020.10.021 33223114 PMC7584496

[114] Rothe K, Feihl S, Schneider J, Wallnofer F, Wurst M, Lukas M, et al. Rates of bacterial co-infections and antimicrobial use in COVID-19 patients: a retrospective cohort study in light of antibiotic stewardship. Eur J Clin Microbiol Infect Dis. 2021;40(4):859–69. 10.1007/s10096-020-04063-8 33140176 PMC7605734

[115] Segrelles-Calvo G, Araujo GRS, Llopis-Pastor E, Carrillo J, Hernandez-Hernandez M, Rey L, et al. Prevalence of opportunistic invasive aspergillosis in COVID-19 patients with severe pneumonia. Mycoses. 2021;64(2):144–51. 10.1111/myc.13219 33217071 PMC7753478

[116] Sharifipour E, Shams S, Esmkhani M, Khodadadi J, Fotouhi-Ardakani R, Koohpaei A, et al. Evaluation of bacterial co-infections of the respiratory tract in COVID-19 patients admitted to ICU. BMC infectious diseases. 2020;20(1):646. 10.1186/s12879-020-05374-z 32873235 PMC7461753

[117] Sogaard KK, Baettig V, Osthoff M, Marsch S, Leuzinger K, Schweitzer M, et al. Community-acquired and hospital-acquired respiratory tract infection and bloodstream infection in patients hospitalized with COVID-19 pneumonia. J Intensive Care. 2021;9(1):10. 10.1186/s40560-021-00526-y 33461613 PMC7812551

[118] Soriano MC, Vaquero C, Ortiz-Fernandez A, Caballero A, Blandino-Ortiz A, de Pablo R. Low incidence of co-infection, but high incidence of ICU-acquired infections in critically ill patients with COVID-19. The Journal of infection. 2021;82(2):e20–e1. 10.1016/j.jinf.2020.09.010 32956729 PMC7501527

[119] Tang ML, Li YQ, Chen X, Lin H, Jiang ZC, Gu DL, et al. Co-Infection with Common Respiratory Pathogens and SARS-CoV-2 in Patients with COVID-19 Pneumonia and Laboratory Biochemistry Findings: A Retrospective Cross-Sectional Study of 78 Patients from a Single Center in China. Med Sci Monit. 2021;27:e929783. 10.12659/MSM.929783 33388738 PMC7789049

[120] Torrego A, Pajares V, Fernandez-Arias C, Vera P, Mancebo J. Bronchoscopy in Patients with COVID-19 with Invasive Mechanical Ventilation: A Single-Center Experience. Am J Respir Crit Care Med. 2020;202(2):284–7. 10.1164/rccm.202004-0945LE 32412787 PMC7365381

[121] Townsend L, Hughes G, Kerr C, Kelly M, O’Connor R, Sweeney E, et al. Bacterial pneumonia coinfection and antimicrobial therapy duration in SARS-CoV-2 (COVID-19) infection. JAC Antimicrob Resist. 2020;2(3):dlaa071. 10.1093/jacamr/dlaa071 32864608 PMC7446659

[122] Verroken A, Scohy A, Gerard L, Wittebole X, Collienne C, Laterre PF. Co-infections in COVID-19 critically ill and antibiotic management: a prospective cohort analysis. Crit Care. 2020;24(1):410. 10.1186/s13054-020-03135-7 32646494 PMC7347259

[123] Wang L, Amin AK, Khanna P, Aali A, McGregor A, Bassett P, et al. An observational cohort study of bacterial co-infection and implications for empirical antibiotic therapy in patients presenting with COVID-19 to hospitals in North West London. Journal of Antimicrobial Chemotherapy. 2020. 10.1093/jac/dkaa475 PMC7717240 33185241

[124] Wei L, Gao X, Chen S, Zeng W, Wu J, Lin X, et al. Clinical Characteristics and Outcomes of Childbearing-Age Women With COVID-19 in Wuhan: Retrospective, Single-Center Study. Journal of Medical Internet Research. 2020;22(8):e19642. 10.2196/19642 32750000 PMC7446716

[125] White PL, Dhillon R, Cordey A, Hughes H, Faggian F, Soni S, et al. A national strategy to diagnose COVID-19 associated invasive fungal disease in the ICU. Clinical Infectious Diseases. 2020. 10.1093/cid/ciaa1298 32860682 PMC7499527

[126] Wu Q, Xing Y, Shi L, Li W, Gao Y, Pan S, et al. Coinfection and Other Clinical Characteristics of COVID-19 in Children. Pediatrics. 2020;146(1). 10.1542/peds.2020-0961 32376725

[127] Xia P, Wen Y, Duan Y, Su H, Cao W, Xiao M, et al. Clinicopathological Features and Outcomes of Acute Kidney Injury in Critically Ill COVID-19 with Prolonged Disease Course: A Retrospective Cohort. J Am Soc Nephrol. 2020;31(9):2205–21. 10.1681/ASN.2020040426 32826326 PMC7461691

[128] Xu J, Yang X, Yang L, Zou X, Wang Y, Wu Y, et al. Clinical course and predictors of 60-day mortality in 239 critically ill patients with COVID-19: a multicenter retrospective study from Wuhan, China. Crit Care. 2020;24(1):394. 10.1186/s13054-020-03098-9 32631393 PMC7336107

[129] Xu S, Shao F, Bao B, Ma X, Xu Z, You J, et al. Clinical Manifestation and Neonatal Outcomes of Pregnant Patients With Coronavirus Disease 2019 Pneumonia in Wuhan, China. Open Forum Infect Dis. 2020;7(7):ofaa283. 10.1093/ofid/ofaa283 32743014 PMC7384380

[130] Xu W, Sun NN, Gao HN, Chen ZY, Yang Y, Ju B, et al. Risk factors analysis of COVID-19 patients with ARDS and prediction based on machine learning. Scientific reports. 2021;11(1):2933. 10.1038/s41598-021-82492-x 33536460 PMC7858607

[131] Yao T, Gao Y, Cui Q, Peng B, Chen Y, Li J, et al. Clinical characteristics of a group of deaths with COVID-19 pneumonia in Wuhan, China: a retrospective case series. BMC infectious diseases. 2020;20(1):695. 10.1186/s12879-020-05423-7 32962639 PMC7506806

[132] Yu C, Zhang Z, Guo Y, Shi J, Pei G, Yao Y, et al. Lopinavir/ritonavir is associated with pneumonia resolution in COVID-19 patients with influenza coinfection: A retrospective matched-pair cohort study. J Med Virol. 2021;93(1):472–80. 10.1002/jmv.26260 32621621 PMC7361199

[133] Yue H, Zhang M, Xing L, Wang K, Rao X, Liu H, et al. The epidemiology and clinical characteristics of co-infection of SARS-CoV-2 and influenza viruses in patients during COVID-19 outbreak. Journal of Medical Virology. 2020;92(11):2870–3. 10.1002/jmv.26163 32530499 PMC7307028

[134] Yusuf E, Vonk A, van den Akker JPC, Bode L, Sips GJ, Rijnders BJA, et al. Frequency of Positive Aspergillus Tests in COVID-19 Patients in Comparison to Other Patients with Pulmonary Infections Admitted to the ICU. Journal of Clinical Microbiology. 2020. 10.1128/JCM.02278-20 PMC8106735 33277340

[135] Zhang C, Gu J, Chen Q, Deng N, Li J, Huang L, et al. Clinical and epidemiological characteristics of pediatric SARS-CoV-2 infections in China: A multicenter case series. PLoS Medicine. 2020;17(6):e1003130. 10.1371/journal.pmed.1003130 32544155 PMC7297312

[136] Zhang H, Zhang Y, Wu J, Li Y, Zhou X, Li X, et al. Risks and features of secondary infections in severe and critical ill COVID-19 patients. Emerg Microbes Infect. 2020;9(1):1958–64. 10.1080/22221751.2020.1812437 32815458 PMC8284966

[137] Joseph C, Togawa Y, Shindo N. Bacterial and viral infections associated with influenza. Influenza Other Respir Viruses. 2013;7 Suppl 2:105–13. 10.1111/irv.12089 24034494 PMC5909385

[138] Klein EY, Monteforte B, Gupta A, Jiang W, May L, Hsieh YH, et al. The frequency of influenza and bacterial coinfection: a systematic review and meta-analysis. Influenza Other Respir Viruses. 2016;10(5):394–403. 10.1111/irv.12398 27232677 PMC4947938

[139] Wongsurakiat P, Tulatamakit S. Clinical pulmonary infection score and a spot serum procalcitonin level to guide discontinuation of antibiotics in ventilator-associated pneumonia: a study in a single institution with high prevalence of nonfermentative gram-negative bacilli infection. Therapeutic Advances in Respiratory Disease. 2018;12:1753466618760134. 10.1177/1753466618760134 PMC5941665 29506460

[140] Rawson TM, Wilson RC, Holmes A. Understanding the role of bacterial and fungal infection in COVID-19. Clin Microbiol Infect. 2021;27(1):9–11. 10.1016/j.cmi.2020.09.025 32979569 PMC7546203

[141] Garcia-Vidal C, Sanjuan G, Moreno-Garcia E, Puerta-Alcalde P, Garcia-Pouton N, Chumbita M, et al. Incidence of co-infections and superinfections in hospitalized patients with COVID-19: a retrospective cohort study. Clinical Microbiology and Infection. 2021;27(1):83–8. 10.1016/j.cmi.2020.07.041 32745596 PMC7836762

[142] Gagnier JJ, Kienle G, Altman DG, Moher D, Sox H, Riley D, et al. The CARE Guidelines: Consensus-based Clinical Case Reporting Guideline Development. Glob Adv Health Med. 2013;2(5):38–43. 10.7453/gahmj.2013.008 24416692 PMC3833570

