# Supplementary figures and images for: Prevalence and outcomes of co-infection and superinfection with SARS-CoV-2 and other pathogens: A systematic review and meta-analysis
Source: PLoS One. 2021 May 6;16(5):e0251170. doi: 10.1371/journal.pone.0251170 (PMC8101968; doi:10.1371/journal.pone.0251170)

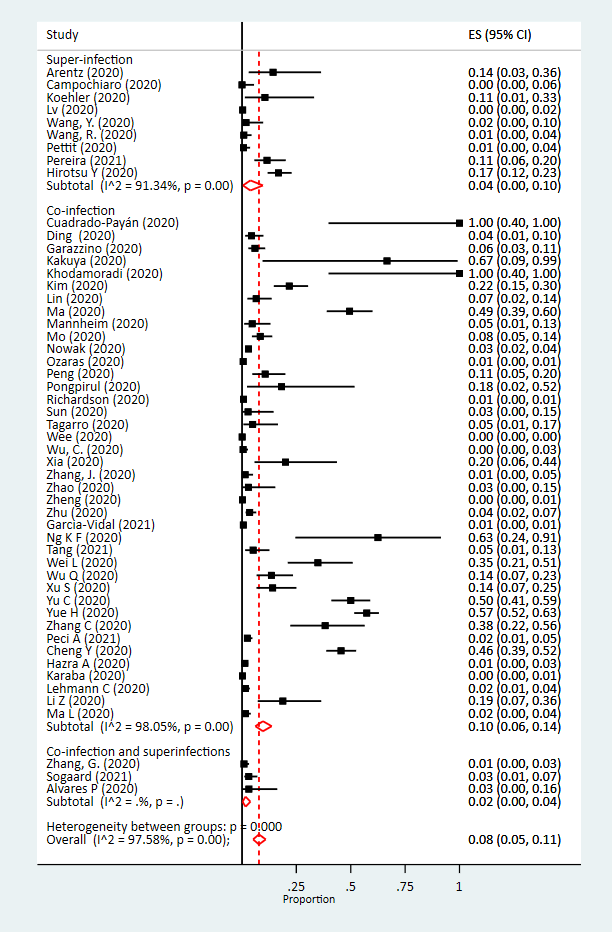

Supplement: S1 Fig — (TIF) [file pone.0251170.s001.tif]

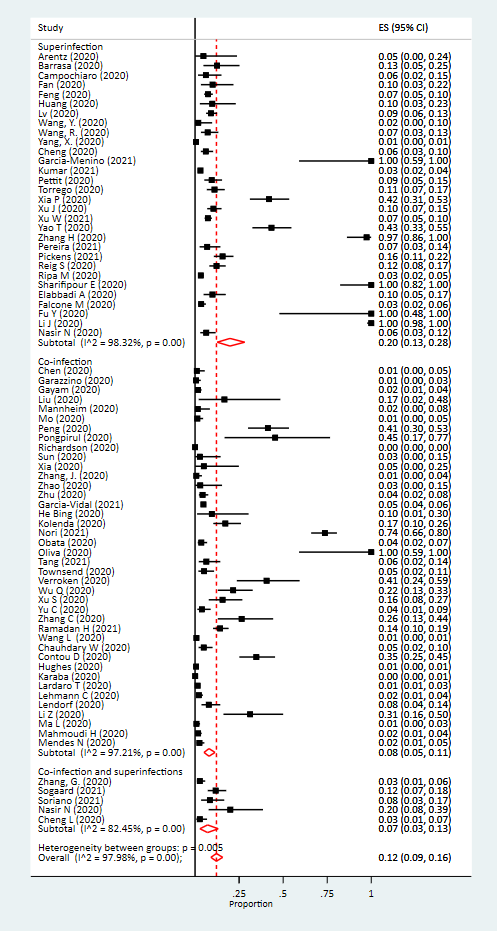

Supplement: S2 Fig — (TIF) [file pone.0251170.s002.tif]

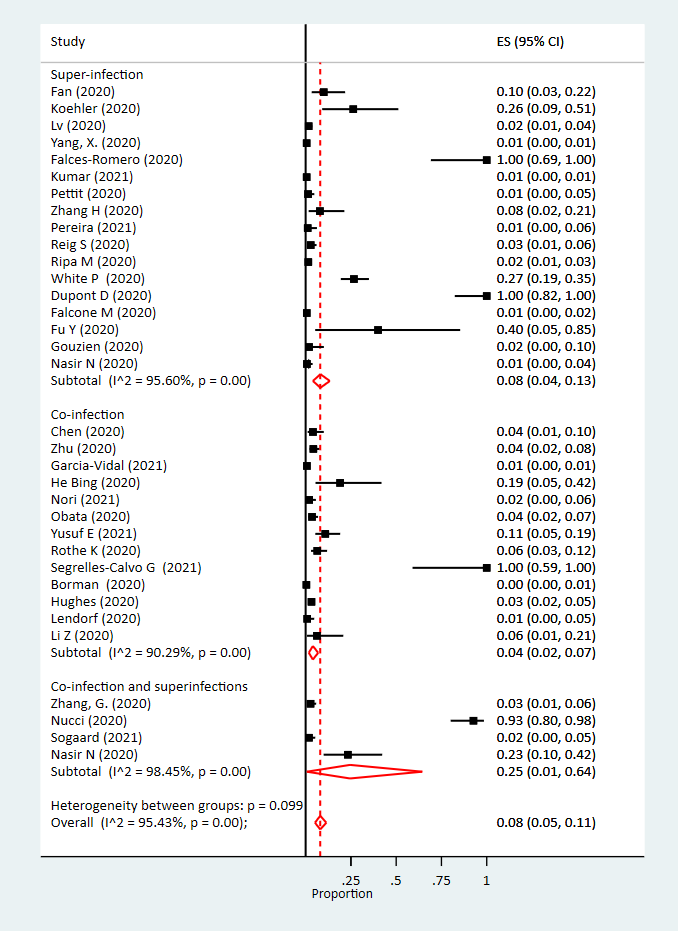

Supplement: S3 Fig — (TIF) [file pone.0251170.s003.tif]
