# Supplementary material for: Prevalence and outcomes of co-infection and superinfection with SARS-CoV-2 and other pathogens: A systematic review and meta-analysis
Source: PLoS One. 2021 May 6;16(5):e0251170. doi: 10.1371/journal.pone.0251170 (PMC8101968; doi:10.1371/journal.pone.0251170)
Supplement: S2 File — (PDF) [file pone.0251170.s005.pdf]

Database searched	Date searched	Results
PubMed	6/11/2020	**526**
Scopus	6/11/2020	**619**
Web of Science	6/11/2020	**304**
CINAHL Plus with Full-Text (Ebsco)	6/11/2020	**253**
Cochrane Central Register of Controlled Trials (Wiley)	6/11/2020	**118**
Hand Searching	6/16/2020	**20**
Total		**1,820**
After Covidence de-duplication		**1,309**

Database searched	Date searched	Results
PubMed	2/8/2021	**3,320**
Scopus	2/8/2021	**3,763**
Web of Science	2/8/2021	**2,385**
CINAHL Plus with Full-Text (Ebsco)	2/8/2021	**463**
Cochrane Central Register of Controlled Trials (Wiley)	2/8/2021	**2,686**
Total		**12,617**
After Covidence de-duplication		**5,320**

Working Search Methods:

The review team collaborated with a research librarian (LAC) to develop and execute a comprehensive search of the literature. This search combined controlled vocabulary and title/abstract terms related to the care of critically ill Covid-19 patients with co-infections. The Covid-19 terms were adapted from search strategies developed by expert searchers and posted on the Medical Library Association (MLA) and Covid Expert Search Strategies websites. The Covid-19 search terms were designed to retrieve studies starting in October 2019, prior to the identification of the identified case of Covid-19. The search was created in PubMed and translated in the following databases: Scopus, Web of Science, CINAHL Plus with Full-Text (Ebsco), Cochrane Central Register of Controlled Trials (Wiley). 20 articles were identified through hand searching of the literature and were added into Covidence for screening. All searches were performed on June 11, 2020. An updated search with new 2021 Covid-19 MeSH terms was run on February 8, 2021 and included records from June 12, 2021 to present.
An English language filter was applied to the results: No other publication type or date filters were applied. Results were downloaded to a citation management software (EndNote), and then imported into Covidence for de-duplication. Unique records were then screened for independent review by several project team members using a pre-determined inclusion/exclusion criteria.

Working Searches (June 11, 2020)
Note: Covid-19 terms adapted from:

1. MLA Covid 19 Literature Searches: https://www.mlanet.org/p/cm/ld/fid=1713
2. OVID Covid-19 Expert Search Strategies: https://tools.ovid.com/coronavirus/

PubMed
Search Date: 6/11/2020
526 results

((“Coronavirus”[mesh] OR “Coronavirus Infections”[mesh] OR coronavir*[tw] OR “corona vir*”[tw] OR OC43[all] OR NL63[all] OR 229E[all] OR HKU1[all] OR HCoV*[tw] OR nCoV*[tw] OR covid*[tw] OR SARS*[tw] OR “Severe Acute Respiratory Syndrome Coronavir*”[tw] OR ((pneumonia[mesh] OR pneumonia*[tw] OR “Influenza, Human”[mesh] OR influenza*[tw] OR flu[tw]) AND (wuhan[tiab] OR hubei[tiab])) AND 2019/10[pdat]:2030[pdat])) OR ("COVID-19"[Supplementary Concept] OR "Severe Acute Respiratory Syndrome Coronavirus 2"[Supplementary Concept] OR “2019 nCoV”[tw] OR nCoV19[tw] OR “nCoV 19”[tw] OR “SARS CoV2"[tw] OR “SARS CoV 2”[tw] OR SARSCoV2[tw]OR “SARSCoV 2”[tw] OR “SARS coronavirus2”[tw] OR “SARS coronavirus 2”[tw] OR “SARS like coronavir*”[tw] OR “severe acute respiratory syndrome coronavirus 2”[tw] OR “coronavirus 19”[tw] OR covid19[tw] OR “covid 19”[tw] OR covid2019[tw] OR “covid 2019”[tw] OR nCoV2019[tw] OR “nCoV 2019”[tw] OR (“2019 novel”[tw] AND (coronavir* OR “corona vir*”[tw] OR CoV[tw] OR nCOV[tw] OR covid[tw]))) AND (“Coinfection”[mesh] OR “Superinfection”[mesh] OR superinfect*[tw] OR “super infect*”[tw] OR coinfect*[tw] OR “co infect*”[tw] OR ((bacteria*[tw] OR fungus[tw] OR fungal[tw] OR mycoses[tw] OR secondary[tw] OR concomitant[tw] OR mixed[tw]) AND infect*[tw]) OR “Pneumovirinae”[mesh] OR “Pneumovirus Infections”[mesh] OR pneumovir*[tw] OR "Respiratory Syncytial Viruses"[Mesh] OR “respiratory syncytial virus*”[tw] OR RSV[tw] OR “Metapneumovirus”[mesh] OR metapneumovir*[tw] OR “meta pneumovir*”[tw] OR HMPV[tw] OR “Influenza, Human”[mesh] OR “Influenzavirus A”[mesh] OR influenza*[tw] OR “respiratory virus*”[tw] OR “Bacterial Infections and Mycoses”[mesh] OR (“upper respiratory”[tw] AND (infection*[tw] OR virus*[tw]))) AND (“Respiration, Artificial”[Mesh] OR (artificial[tw] AND respiration*[tw]) OR oxygen[tw] OR “Oxygen Inhalation Therapy”[mesh] OR “Intensive Care Units”[mesh] OR “intensive care”[tw] OR ICU OR ICUs[tw] OR “Critical Care”[mesh] OR “critical care”[tw] OR critical*[tiab] OR ventilat*[tw] OR intubat*[tw] OR outcome*[tw] OR “Nursing Homes”[mesh] OR “Subacute Care”[mesh] OR “nursing home*”[tw] OR “skilled nursing”[tw] OR “intermediate care”[tw] OR “Patient Discharge”[mesh] OR “Mortality”[mesh] OR “Mortality”[subheading] OR mortalit*[tw] OR “Morbidity”[mesh] OR death*[tw] OR morbidit*[tw] OR (patient*[tw] AND discharg*[tw])) AND English[filter]

Scopus
Search Date: 6/11/2020
619 results

((TITLE-ABS-KEY(coronavir* OR "corona vir*" OR OC43 OR NL63 OR 229E OR HKU1 OR HCoV* OR ncov* OR covid* OR sars* OR "Severe Acute Respiratory Syndrome")) OR (TITLE-ABS-KEY((pneumonia* OR influenza* OR flu) AND (wuhan OR hubei))) AND ((PUBDATETXT (“october 2019” OR "november 2019" OR "december 2019")) OR (PUBYEAR > 2019))) OR (TITLE-ABS-KEY("Severe Acute Respiratory Syndrome Coronavirus 2" OR "2019 ncov" OR ncov19 OR "ncov 19" OR "2019 novel CoV" OR "sars cov2" OR "SARS CoV 2" OR SARSCoV2 OR "SARSCoV 2" OR "SARS coronavirus2" OR "SARS coronavirus 2" OR "SARS like coronavir*" OR "coronavirus 19" OR covid19 OR "covid 19" OR covid2019 OR "covid 2019" OR nCoV2019 OR "nCoV 2019")) AND (TITLE-ABS-KEY (coinfect* OR "co infect*" OR superinfect* OR “super infect*”)) OR (TITLE-ABS-KEY ((bacteria* OR fungus OR fungal OR mycoses OR secondary OR concomitant OR mixed) AND infect*)) OR (TITLE-ABS-KEY (pneumovir* OR "respiratory syncytial virus*" OR rsv OR metapneumovir* OR “meta pneumovir*” OR hmpv OR influenza* OR (respiratory W/2 virus*))) OR (TITLE-ABS-KEY ((upper W/3 respiratory) AND (infection* OR virus*))) AND TITLE-ABS-KEY((artificial AND respiration*) OR oxygen OR "intensive care" OR ICU OR ICUs OR "critical care" OR ventilat* OR intubat* OR outcome* OR "nursing home*" OR "skilled nursing" OR “subacute care” OR "intermediate care" OR mortalit* OR death* OR morbidit* OR (patient* AND discharg*)) OR TITLE-ABS(critical*) AND (LIMIT-TO (LANGUAGE , "English"))

Web of Science Core Collection
Search Date: 6/11/2020
304 results

(((TS=(coronavir* OR "corona vir*" OR OC43 OR NL63 OR 229E OR HKU1 OR HCoV* OR ncov* OR covid* OR sars* OR "Severe Acute Respiratory
Syndrome") OR TS=((pneumonia* OR influenza* OR flu) AND (wuhan OR
hubei))) AND PY=(2019 OR 2020)) OR (TS=("Severe Acute Respiratory Syndrome Coronavirus 2" OR "2019 ncov" OR ncov19 OR "ncov 19" OR "2019 novel CoV" OR "sars cov2" OR "SARS CoV 2" OR SARSCoV2 OR "SARSCoV 2" OR "SARS coronavirus2" OR "SARS coronavirus 2" OR "SARS like coronavir*" OR "coronavirus 19" OR covid19 OR "covid 19" OR covid2019 OR "covid 2019" OR nCoV2019 OR "nCoV 2019"))) AND ((TS=(coinfect* OR "co infect*" OR superinfect* OR “super infect*”)) OR (TS=(bacteria* OR fungus OR fungal OR mycoses OR secondary OR concomitant OR mixed
) AND TS=(infect*)) OR (TS=(pneumovir* OR "respiratory syncytial virus*" OR rsv OR metapneumovir* OR “meta pneumovir*” OR hmpv OR influenza* OR (respiratory NEAR/2 virus*))) OR (TS=("upper respiratory" AND (infection* OR virus*)))) AND ((TS=(artificial

AND respiration*)) OR (TS=(oxygen OR "intensive care" OR ICU OR ICUs OR "critical care" OR ventilat* OR intubat* OR outcome* OR "nursing home*" OR "skilled nursing" OR “subacute care” OR "intermediate care" OR mortalit* OR death* OR morbidit*)) OR (TS=(patient AND discharg*)) OR (TI=(critical*)) OR (AB=(critical*))) AND (LA=(English))

CINAHL Plus
Search Date: 6/11/2020
253 Results

Search History

S30	S25 AND S26 AND S28 Limiters - English
S29	S25 AND S26 AND S28
S28	S15 OR S16 OR S17 OR S18 OR S19 OR S20 OR S21 OR S22 OR S23 OR S24 OR S27
S27	TX ((artificial AND respiration*) OR oxygen OR "intensive care" OR ICU OR ICUs OR "critical care" OR ventilat* OR intubat* OR outcome* OR "nursing home*" OR "skilled nursing" OR “subacute care” OR "intermediate care" OR mortalit* OR death* OR morbidit* OR (patient* AND discharg*)) OR TI critical* OR AB critical*
S26	S8 OR S9 OR S10 OR S11 OR S12 OR S13 OR S14
S25	S6 OR S7
S24	(MW "mo")
S23	(MH "Morbidity+")
S22	(MH "Mortality+")
S21	(MH "Patient Discharge+")
S20	(MH "Subacute Care")
S19	(MH "Nursing Homes+")
S18	(MH "Critical Care+")
S17	(MH "Intensive Care Units+")

S16	(MH "Oxygen Therapy+")
S15	(MH "Respiration, Artificial+")
S14	TX (upper N3 respiratory) AND (infection* OR virus*)
S13	TX (pneumovir* OR "respiratory syncytial virus*" OR RSV OR metapneumovir* OR "meta pneumovir*" OR HMPV OR influenza* OR "respiratory virus*")
S12	(MH "Bacterial Infections+") OR (MH "Mycoses+")
S11	(MH "Influenza, Human+") OR (MH "Influenzavirus A+")
S10	(MH "Respiratory Syncytial Viruses") OR (MH "Respiratory Syncytial Virus Infections")
S9	TX (bacteria* OR fungus OR fungal OR mycoses OR secondary OR concomitant OR mixed) AND infect*
S8	(MH "Coinfection") OR (MH "superinfection") OR (TX coinfect* OR "co infect* OR superinfect* OR "super infect*")
S7	TX "Severe Acute Respiratory Syndrome Coronavirus 2" OR "2019 ncov" OR ncov19 OR "ncov 19" OR "2019 novel CoV" OR "sars cov2" OR "SARS CoV 2" OR SARSCoV2 OR "SARSCoV 2" OR "SARS coronavirus2" OR "SARS coronavirus 2" OR "SARS like coronavir*" OR "coronavirus 19" OR covid19 OR "covid 19" OR covid2019 OR "covid 2019" OR nCoV2019 OR "nCoV 2019"
S6	S1 OR S2 OR S3 OR S4 Limiters - Published Date: 20191001-
S5	S1 OR S2 OR S3 OR S4
S4	(MH "Pneumonia+" OR MH "Influenza, Human+" OR TX (pneumonia* OR influenza* OR flu))

	AND (TI (wuhan OR hubei) OR AB (wuhan OR hubei))
S3	TX (coronavir* OR "corona vir*" OR HCoV* OR ncov* OR covid* OR sars* OR "Severe Acute Respiratory Syndrome") OR (OC43 OR NL63 OR 229E OR HKU1)
S2	(MH "Coronavirus Infections+")
S1	(MH "Coronavirus+")

Cochrane Central Register of Controlled Trials (CENTRAL Issue 6 of 12, June 2020)
Search Date: 6/11/2020
Search Name: Musuuza RR covid-19 and co-infection
118 Results

ID Search

#1 MeSH descriptor: [Coronavirus] explode all trees

#2 MeSH descriptor: [Coronavirus Infections] explode all trees

#3 ((coronavir* OR "corona vir*" OR HCoV* OR ncov* OR covid* OR sars* OR "Severe Acute Respiratory Syndrome Coronavir*")):ti,ab,kw

#4 ((OC43 OR NL63 OR 229E OR HKU1))

#5 #1 OR #2 OR #3 OR #4

#6 MeSH descriptor: [Pneumonia] explode all trees

#7 MeSH descriptor: [Influenza, Human] explode all trees

#8 (pneumonia* OR influenza* OR flu):ti,ab,kw

#9 #6 OR #7 OR #8

#10 (wuhan OR hubei):ti

#11 (wuhan OR hubei):ab

#12 #10 OR #11

#13 #9 AND #12

#14 #5 OR #13 with Cochrane Library publication date Between Oct 2019 and Dec 2020

#15 ("Severe Acute Respiratory Syndrome Coronavirus 2" OR "2019 ncov" OR ncov19 OR "ncov 19" OR "2019 novel CoV" OR "sars cov2" OR "SARS CoV 2" OR SARSCoV2 OR "SARSCoV 2" OR "SARS coronavirus2" OR "SARS coronavirus 2" OR "SARS like coronavir*" OR "coronavirus 19" OR covid19 OR "covid 19" OR covid2019 OR "covid 2019" OR nCoV2019 OR "nCoV 2019"):ti,ab,kw

#16 #14 OR #15

#17 MeSH descriptor: [Coinfection] explode all trees

#18 (coinfect* OR "co infect*"):ti,ab,kw

#19 MeSH descriptor: [Superinfection] explode all trees

#20 (superinfect* OR "super infect*"):ti,ab,kw

#21 ((bacteria* OR fungus OR fungal OR mycoses OR secondary OR concomitant OR mixed) AND infect*):ti,ab,kw

#22 MeSH descriptor: [Pneumovirinae] explode all trees

#23 MeSH descriptor: [Respiratory Syncytial Viruses] explode all trees

#24 MeSH descriptor: [Pneumovirus Infections] explode all trees

#25 MeSH descriptor: [Metapneumovirus] explode all trees

#26 MeSH descriptor: [Influenza, Human] explode all trees

#27 MeSH descriptor: [Influenzavirus A] explode all trees

#28 MeSH descriptor: [Bacterial Infections and Mycoses] explode all trees

#29 (pneumovir* OR "respiratory syncytial virus*" OR rsv OR metapneumovir* OR “meta pneumovir*” OR hmpv OR influenza* OR (respiratory NEAR/2 virus*)):ti,ab,kw

#30 ((upper NEAR/3 respiratory) AND (infection* OR virus*)):ti,ab,kw

#31 #17 OR #18 OR #19 OR #20 OR #21 OR #22 OR #23 OR #24 OR #25 OR #26 OR #27 OR #28 OR #29 OR #30

#32 MeSH descriptor: [Respiration, Artificial] explode all trees

#33 (artificial AND respiration*):ti,ab,kw

#34 MeSH descriptor: [Oxygen Inhalation Therapy] explode all trees

#35 MeSH descriptor: [Intensive Care Units] explode all trees

#36 MeSH descriptor: [Critical Care] explode all trees

#37 MeSH descriptor: [Nursing Homes] explode all trees

#38 MeSH descriptor: [Patient Discharge] explode all trees

#39 MeSH descriptor: [Subacute Care] explode all trees

#40 MeSH descriptor: [Mortality] explode all trees

#41 MeSH descriptor: [Morbidity] explode all trees

#42 MeSH descriptor: [] explode all trees and with qualifier(s): [mortality - MO]

#43 (oxygen OR "intensive care" OR ICU OR ICUs OR "critical care" OR ventilat* OR intubat* OR outcome* OR "nursing home*" OR "subacute care" OR "skilled nursing" OR "intermediate care" OR mortalit* OR death* OR morbidit* OR (patient* AND discharg*)):ti,ab,kw

#44 (critical*):ti

#45 (critical*):ab

#46 #32 OR #33 OR #34 OR #35 OR #36 OR #37 OR #38 OR #39 OR #40 OR #41 OR #42 OR #43 OR #44 OR #45

#47 #16 AND #31 AND #46 in Trials

Working Searches (February 8, 2021)

PubMed
Re-run search Date: 2/8/21
3320 results

((“Coronavirus”[mesh] OR “Coronavirus Infections”[mesh] OR coronavir*[tw] OR “corona vir*”[tw] OR OC43[all] OR NL63[all] OR 229E[all] OR HKU1[all] OR HCoV*[tw] OR nCoV*[tw] OR covid*[tw] OR SARS*[tw] OR “Severe Acute Respiratory Syndrome Coronavir*”[tw] OR ((“Pneumonia”[mesh] OR pneumonia*[tw] OR “Influenza, Human”[mesh] OR influenza*[tw] OR flu[tw]) AND (wuhan[tiab] OR hubei[tiab])) AND 2019/10[pdat]:2030[pdat])) OR ("COVID-19"[Mesh] OR "SARS-CoV-2"[Mesh] OR “2019 nCoV”[tw] OR nCoV19[tw] OR “nCoV 19”[tw] OR “SARS CoV2"[tw] OR “SARS CoV 2”[tw] OR SARSCoV2[tw]OR “SARSCoV 2”[tw] OR “SARS coronavirus2”[tw] OR “SARS coronavirus 2”[tw] OR “SARS like coronavir*”[tw] OR “severe acute respiratory syndrome coronavirus 2”[tw] OR “coronavirus 19”[tw] OR covid19[tw] OR “covid 19”[tw] OR covid2019[tw] OR “covid 2019”[tw] OR nCoV2019[tw] OR “nCoV 2019”[tw] OR (“2019 novel”[tw] AND (coronavir* OR “corona vir*”[tw] OR CoV[tw] OR nCOV[tw] OR covid[tw]))) AND (“Coinfection”[mesh] OR “Superinfection”[mesh] OR superinfect*[tw] OR “super infect*”[tw] OR coinfect*[tw] OR “co infect*”[tw] OR ((bacteria*[tw] OR fungus[tw] OR fungal[tw] OR mycoses[tw] OR secondary[tw] OR concomitant[tw] OR mixed[tw]) AND infect*[tw]) OR “Pneumovirinae”[mesh] OR “Pneumovirus Infections”[mesh] OR pneumovir*[tw] OR "Respiratory Syncytial Viruses"[Mesh] OR “respiratory syncytial

virus*”[tw] OR RSV[tw] OR “Metapneumovirus”[mesh] OR metapneumovir*[tw] OR “meta pneumovir*”[tw] OR HMPV[tw] OR “Influenza, Human”[mesh] OR “Influenzavirus A”[mesh] OR influenza*[tw] OR “respiratory virus*”[tw] OR “Bacterial Infections and Mycoses”[mesh] OR (“upper respiratory”[tw] AND (infection*[tw] OR virus*[tw]))) AND (“Respiration, Artificial”[Mesh] OR (artificial[tw] AND respiration*[tw]) OR oxygen[tw] OR “Oxygen Inhalation Therapy”[mesh] OR “Intensive Care Units”[mesh] OR “intensive care”[tw] OR ICU[tw] OR ICUs[tw] OR “Critical Care”[mesh] OR “critical care”[tw] OR critical*[tiab] OR ventilat*[tw] OR intubat*[tw] OR outcome*[tw] OR “Nursing Homes”[mesh] OR “Subacute Care”[mesh] OR “nursing home*”[tw] OR “skilled nursing”[tw] OR “intermediate care”[tw] OR “Patient Discharge”[mesh] OR “Mortality”[mesh] OR “Mortality”[subheading] OR mortalit*[tw] OR “Morbidity”[mesh] OR death*[tw] OR morbidit*[tw] OR (patient*[tw] AND discharg*[tw])) AND English[filter] AND (("2020/06/11"[CRDT] : "3000"[CRDT]) OR ("2020/06/11"[EDAT] : "3000"[EDAT]) OR ("2020/06/11"[MHDA] : "3000"[MHDA]))

Scopus
Re-run Search Date: 2/8/2021
3763 results
((TITLE-ABS-KEY(coronavir* OR "corona vir*" OR OC43 OR NL63 OR 229E OR HKU1 OR HCoV* OR ncov* OR covid* OR sars* OR "Severe Acute Respiratory Syndrome")) OR (TITLE-ABS-KEY((pneumonia* OR influenza* OR flu) AND (wuhan OR hubei))) AND ((PUBDATETXT (“october 2019” OR "november 2019" OR "december 2019")) OR (PUBYEAR > 2019))) OR (TITLE-ABS-KEY("Severe Acute Respiratory Syndrome Coronavirus 2" OR "2019 ncov" OR ncov19 OR "ncov 19" OR "2019 novel CoV" OR "sars cov2" OR "SARS CoV 2" OR SARSCoV2 OR "SARSCoV 2" OR "SARS coronavirus2" OR "SARS coronavirus 2" OR "SARS like coronavir*" OR "coronavirus 19" OR covid19 OR "covid 19" OR covid2019 OR "covid 2019" OR nCoV2019 OR "nCoV 2019")) AND (TITLE-ABS-KEY (coinfect* OR "co infect*" OR superinfect* OR “super infect*”)) OR (TITLE-ABS-KEY ((bacteria* OR fungus OR fungal OR mycoses OR secondary OR concomitant OR mixed) AND infect*)) OR (TITLE-ABS-KEY (pneumovir* OR "respiratory syncytial virus*" OR rsv OR metapneumovir* OR “meta pneumovir*” OR hmpv OR influenza* OR (respiratory W/2 virus*))) OR (TITLE-ABS-KEY ((upper W/3 respiratory) AND (infection* OR virus*))) AND ((TITLE-ABS-KEY((artificial AND respiration*) OR oxygen OR "intensive care" OR ICU OR ICUs OR "critical care" OR ventilat* OR intubat* OR outcome* OR "nursing home*" OR "skilled nursing" OR “subacute care” OR "intermediate care" OR mortalit* OR death* OR morbidit* OR (patient* AND discharg*))) OR (TITLE-ABS(critical*))) AND LOAD-DATE AFT 20200611 AND (LIMIT-TO (LANGUAGE , "English"))

Web of Science (SCI-EXPANDED, SSCI, A&HCI, CPCI-S, CPCI-SSH, BKCI-S, BKCI-SSH, ESCI)
Search Date: 2/8/2021
2385 Results

(((TS=(coronavir* OR "corona vir*" OR OC43 OR NL63 OR 229E OR HKU1 OR HCoV* OR ncov* OR covid* OR sars* OR "Severe Acute Respiratory Syndrome") OR TS=((pneumonia* OR influenza* OR flu) AND (wuhan OR hubei))) AND PY=(2019 OR 2020)) OR (TS=("Severe Acute Respiratory Syndrome Coronavirus 2" OR "2019 ncov" OR ncov19 OR "ncov 19" OR "2019 novel CoV" OR "sars cov2" OR "SARS CoV 2" OR SARSCoV2 OR "SARSCoV 2" OR "SARS coronavirus2" OR "SARS coronavirus 2" OR "SARS like coronavir*" OR "coronavirus 19" OR covid19 OR "covid 19" OR covid2019 OR "covid 2019" OR nCoV2019 OR "nCoV 2019"))) AND ((TS=(coinfect* OR "co infect*" OR superinfect* OR “super infect*”)) OR (TS=(bacteria* OR fungus OR fungal OR mycoses OR secondary OR concomitant OR mixed) AND TS=(infect*)) OR (TS=(pneumovir* OR "respiratory syncytial virus*" OR rsv OR metapneumovir* OR “meta pneumovir*” OR hmpv OR influenza* OR (respiratory NEAR/2 virus*))) OR (TS=("upper respiratory" AND (infection* OR virus*)))) AND ((TS=(artificial AND respiration*)) OR (TS=(oxygen OR "intensive care" OR ICU OR ICUs OR "critical care" OR ventilat* OR intubat* OR outcome* OR "nursing home*" OR "skilled nursing" OR “subacute care” OR "intermediate care" OR mortalit* OR death* OR morbidit*)) OR (TS=(patient AND discharg*)) OR (TI=(critical*)) OR (AB=(critical*))) AND (LA=(English))

CINAHL Plus
Search Date: 2/8/21
463 Results

Search History

S30	S25 AND S26 AND S28 Limiters - English
S29	S25 AND S26 AND S28
S28	S15 OR S16 OR S17 OR S18 OR S19 OR S20 OR S21 OR S22 OR S23 OR S24 OR S27
S27	((artificial AND respiration*) OR oxygen OR "intensive care" OR ICU OR ICUs OR "critical care" OR ventilat* OR intubat* OR outcome* OR "nursing home*" OR "skilled nursing" OR “subacute care” OR "intermediate care" OR mortalit* OR death* OR morbidit* OR (patient* AND discharg*)) OR (TI critical* OR AB critical*)
S26	S8 OR S9 OR S10 OR S11 OR S12 OR S13 OR S14

S25	S6 OR S7
S24	(MW "mo")
S23	(MH "Morbidity+")
S22	(MH "Mortality+")
S21	(MH "Patient Discharge+")
S20	(MH "Subacute Care")
S19	(MH "Nursing Homes+")
S18	(MH "Critical Care+")
S17	(MH "Intensive Care Units+")
S16	(MH "Oxygen Therapy+")
S15	(MH "Respiration, Artificial+")
S14	(upper N3 respiratory) AND (infection* OR virus*)
S13	(pneumovir* OR "respiratory syncytial virus*" OR RSV OR metapneumovir* OR "meta pneumovir*" OR HMPV OR influenza* OR "respiratory virus*")
S12	(MH "Bacterial Infections+") OR (MH "Mycoses+")
S11	(MH "Influenza, Human+") OR (MH "Influenzavirus A+")
S10	(MH "Respiratory Syncytial Viruses") OR (MH "Respiratory Syncytial Virus Infections")
S9	(bacteria* OR fungus OR fungal OR mycoses OR secondary OR concomitant OR mixed) AND infect*
S8	(MH "Coinfection") OR (MH "superinfection") OR (coinfect* OR "co infect* OR superinfect* OR "super infect*")

S7	"Severe Acute Respiratory Syndrome Coronavirus 2" OR "2019 ncov" OR ncov19 OR "ncov 19" OR "2019 novel CoV" OR "sars cov2" OR "SARS CoV 2" OR SARSCoV2 OR "SARSCoV 2" OR "SARS coronavirus2" OR "SARS coronavirus 2" OR "SARS like coronavir*" OR "coronavirus 19" OR covid19 OR "covid 19" OR covid2019 OR "covid 2019" OR nCoV2019 OR "nCoV 2019"
S6	S1 OR S2 OR S3 OR S4 Limiters - Published Date: 20191001-
S5	S1 OR S2 OR S3 OR S4
S4	(MH "Pneumonia+" OR MH "Influenza, Human+" OR (pneumonia* OR influenza* OR flu)) AND (TI (wuhan OR hubei) OR AB (wuhan OR hubei))
S3	(coronavir* OR "corona vir*" OR HCoV* OR ncov* OR covid* OR sars* OR "Severe Acute Respiratory Syndrome") OR (OC43 OR NL63 OR 229E OR HKU1)
S2	(MH "Coronavirus Infections+")
S1	(MH "Coronavirus+")

Cochrane Central Register of Controlled Trials (Wiley)
(CENTRAL Issue 2 of 12 February)
Search Date: 2/8/2021
2686 Results

ID Search

#1 MeSH descriptor: [Coronavirus] explode all trees

#2 MeSH descriptor: [Coronavirus Infections] explode all trees

#3 ((coronavir* OR corona-vir* OR HCoV* OR ncov* OR covid* OR sars* OR Severe-Acute-Respiratory-Syndrome-Coronavir*)):ti,ab,kw

#4 ((OC43 OR NL63 OR 229E OR HKU1))

#5 #1 OR #2 OR #3 OR #4

#6 MeSH descriptor: [Pneumonia] explode all trees

#7 MeSH descriptor: [Influenza, Human] explode all trees

#8 (pneumonia* OR influenza* OR flu):ti,ab,kw

#9 #6 OR #7 OR #8

#10 (wuhan OR hubei):ti

#11 (wuhan OR hubei):ab

#12 #10 OR #11

#13 #9 AND #12

#14 #5 OR #13 with Cochrane Library publication date Between Oct 2019 and Dec 2020

#15 ("Severe Acute Respiratory Syndrome Coronavirus 2" OR "2019 ncov" OR ncov19 OR "ncov 19" OR "2019 novel CoV" OR "sars cov2" OR "SARS CoV 2" OR SARSCoV2 OR "SARSCoV 2" OR "SARS coronavirus2" OR "SARS coronavirus 2" OR SARS-like-coronavir* OR “coronavirus 19” OR covid19 OR "covid 19" OR covid2019 OR "covid 2019" OR nCoV2019 OR "nCoV 2019"):ti,ab,kw

#16 #14 OR #15

#17 MeSH descriptor: [Coinfection] explode all trees

#18 (coinfect* OR "co infect*"):ti,ab,kw

#19 MeSH descriptor: [Superinfection] explode all trees

#20 (superinfect* OR "super infect*"):ti,ab,kw

#21 ((bacteria* OR fungus OR fungal OR mycoses OR secondary OR concomitant OR mixed) AND infect*):ti,ab,kw

#22 MeSH descriptor: [Pneumovirinae] explode all trees

#23 MeSH descriptor: [Respiratory Syncytial Viruses] explode all trees

#24 MeSH descriptor: [Pneumovirus Infections] explode all trees

#25 MeSH descriptor: [Metapneumovirus] explode all trees

#26 MeSH descriptor: [Influenza, Human] explode all trees

#27 MeSH descriptor: [Influenzavirus A] explode all trees

#28 MeSH descriptor: [Bacterial Infections and Mycoses] explode all trees

#29 (pneumovir* OR "respiratory syncytial virus*" OR rsv OR metapneumovir* OR “meta pneumovir*” OR hmpv OR influenza* OR (respiratory NEAR/2 virus*)):ti,ab,kw

#30 ((upper NEAR/3 respiratory) AND (infection* OR virus*)):ti,ab,kw

#31 #17 OR #18 OR #19 OR #20 OR #21 OR #22 OR #23 OR #24 OR #25 OR #26 OR #27 OR #28 OR #29 OR #30

#32 MeSH descriptor: [Respiration, Artificial] explode all trees

#33 (artificial AND respiration*):ti,ab,kw

#34 MeSH descriptor: [Oxygen Inhalation Therapy] explode all trees

#35 MeSH descriptor: [Intensive Care Units] explode all trees

#36 MeSH descriptor: [Critical Care] explode all trees

#37 MeSH descriptor: [Nursing Homes] explode all trees

#38 MeSH descriptor: [Patient Discharge] explode all trees

#39 MeSH descriptor: [Subacute Care] explode all trees

#40 MeSH descriptor: [Mortality] explode all trees

#41 MeSH descriptor: [Morbidity] explode all trees

#42 MeSH descriptor: [] explode all trees and with qualifier(s): [mortality - MO]

#43 (oxygen OR "intensive care" OR ICU OR ICUs OR "critical care" OR ventilat* OR intubat* OR outcome* OR "nursing home*" OR "subacute care" OR "skilled nursing" OR "intermediate care" OR mortalit* OR death* OR morbidit* OR (patient* AND discharg*)):ti,ab,kw

#44 (critical*):ti

#45 (critical*):ab

#46 #32 OR #33 OR #34 OR #35 OR #36 OR #37 OR #38 OR #39 OR #40 OR #41 OR #42 OR #43 OR #44 OR #45

#47 #16 AND #31 AND #46 in Trials
